# Preoperative C-Reactive Protein-to-Albumin Ratio Predicts Recipient and Graft Survival After Deceased-Donor Liver Transplantation

**DOI:** 10.3390/jcm15187063

**Published:** 2026-09-11

**Authors:** David Erren, Franziska A. Meister, Karsten Große, Tony Bruns, Naomi Gestels, Oliver Beetz, Felix Oldhafer, Martin W. von Websky, Thomas Vogel, Florian W. R. Vondran, Iakovos Amygdalos

**Affiliations:** 1 Department of General, Visceral, Pediatric, and Transplantation Surgery, University Hospital RWTH Aachen, Pauwelsstraße 30, 52074 Aachen, Germany; 2 Department of Internal Medicine III, University Hospital RWTH, Pauwelsstraße 30, 52074 Aachen, Germany; 3 Department of Internal Medicine, Maastricht University Medical Centre, 6229 HX Maastricht, The Netherlands

**Keywords:** liver transplantation, C-reactive protein-to-albumin ratio, graft survival, recipient survival

## Abstract

**Background/Objectives**: Recipient-related factors continue to influence long-term outcomes after deceased-donor liver transplantation (DDLT). This study evaluated the prognostic value of the preoperative C-reactive protein-to-albumin ratio (CAR) for recipient survival (RS) and graft survival (GS). **Methods**: Adult patients undergoing DDLT between 2010 and 2023 were retrospectively analyzed. Re-transplantations, split or domino transplants, patients who died within 90 days and patients with early allograft dysfunction were excluded. Primary endpoints were 3-year RS and GS. Receiver operating characteristic analysis was used to determine the optimal CAR cut-off. Survival was assessed using Kaplan–Meier analysis and Cox regression. **Results**: A total of 330 patients were included. Mean RS and GS were 112 months (95%CI 106–119) and 110 months (95%CI 103–116), respectively. Preoperative CAR demonstrated the strongest predictive value for 3-year RS (AUC = 0.70, *p* < 0.001) and GS (AUC = 0.71, *p* < 0.001). A CAR cut-off of 41% provided optimal discrimination. Patients with CAR > 41% had significantly reduced 3-year RS (31 vs. 34 months, *p* < 0.001) and GS (30 vs. 34 months, *p* < 0.001). CAR > 41% was independently associated with increased risk of 3-year mortality (HR 2.101, 95%CI 1.277–3.456, *p* = 0.003) and graft loss (HR 3.666, 95%CI 1.804–7.449, *p* < 0.001). Similar results were observed for 5-year outcomes. **Conclusions**: Elevated preoperative CAR is independently associated with inferior patient and graft survival and may enhance risk stratification after DDLT.

## 1. Introduction

Over the past six decades, orthotopic liver transplantation (OLT) has evolved from a pioneering experimental procedure into the gold standard treatment for patients with end-stage liver disease [1,2]. In 2024, more than 40,000 liver transplantations were performed worldwide, reflecting its widespread adoption and clinical significance [3]. This transformation has been driven by advances in surgical techniques, perioperative management, immunosuppressive therapy, and the refinement of recipient selection criteria [1,4].

Despite these improvements, OLT continues to carry significant clinical risks, including early complications, graft dysfunction, and long-term mortality [5,6,7]. Identifying reliable predictors of post-transplant outcomes remains crucial for optimizing patient selection, postoperative care, and long-term monitoring. Several prognostic tools, such as the model for end-stage liver disease (MELD), balance of risk (BAR), and survival outcomes following liver transplantation (SOFT) scores, have been developed for this purpose [1,8,9]. In addition, specific factors such as postoperative platelet count, recipient body mass index (BMI), and immunologic or demographic variables have been explored as potential outcome predictors [4,10].

Among various biomarkers, the C-reactive protein-to-albumin ratio (CAR) has gained attention for its ability to reflect both systemic inflammation and nutritional status. Previous studies have demonstrated its predictive value for morbidity, early mortality, and graft dysfunction across various patient populations, including oncological and liver disease [11,12,13,14,15]. In our previous work, we assessed the association between preoperative CAR and early postoperative outcomes in liver transplant recipients, suggesting its potential utility in short-term risk stratification [16].

However, the long-term prognostic value of CAR in the context of deceased-donor liver transplantation (DDLT) has not been thoroughly investigated. Given the increasing emphasis on improving long-term survival following OLT, this study aims to evaluate the relationship between preoperative CAR and 3-year patient survival after DDLT. By expanding upon earlier findings, this study seeks to determine whether CAR can serve as a reliable biomarker for longer-term outcome prediction in this high-risk population.

## 2. Materials and Methods

Study Design and Endpoints:

This retrospective study included consecutive adult patients who underwent deceased-donor liver transplantation (DDLT) at the University Hospital RWTH Aachen, Germany, between May 2010 and March 2023. Patients who underwent re-transplantation, split liver transplantation, or domino transplantation were excluded. Patients who passed away within 90 days post-transplantation were also excluded, as they had been included in our previous work on CAR, which investigated short-term morbidity and mortality [16]. Finally, patients who suffered from early allograft dysfunction (EAD), according to the Olthoff criteria [17], were also removed. The primary endpoints of the current study were 3-year recipient survival (RS) and graft survival (GS), while secondary endpoints were 5-year RS and GS. Patients who did not experience the respective endpoint were censored at their last available follow-up. The observation period ended in May 2023. Median follow-up was estimated using the reverse Kaplan–Meier method.

The study was conducted under the ethical approval of the Institutional Review Board of RWTH Aachen University (EK-001/21) and in accordance with the current version of the Declaration of Helsinki, the Declaration of Istanbul, and good clinical practice guidelines (ICH GCP). Informed consent was waived due to the retrospective study design and collection of readily available clinical data.

Data Collection and Clinical Considerations:

Organ allocation was conducted in accordance with German national guidelines, as well as international regulations established by Eurotransplant [18]. The surgical procedures for orthotopic liver transplantation (OLT) were standardized following previously described protocols [19]. Perioperative care and immunosuppressive regimens adhered to internal institutional protocols. Clinical data were retrospectively analyzed, having been sourced from a prospective institutional database and medical records. Post-discharge follow-up was managed at the transplantation outpatient departments or at community-based hepatology units. Serum laboratory parameters, including C-reactive protein (CRP in mg/L) and albumin (Alb in g/dL) levels, were routinely measured on the day of surgery in all transplant recipients. A percentage value for CAR (CRP/Alb × 100 = CAR%) was calculated using CRP and Alb values from the same blood sample. Laboratory values and other parameters were used to calculate labMELD, BAR, and SOFT scores. Recipient survival was defined as time from DDLT to death (from any cause), while GS was defined as time from DDLT to death or graft loss (e.g., date of re-transplantation).

Statistical analysis:

Data were reported as median and interquartile range for continuous variables or absolute and relative frequencies for categorical and ordinal variables. The predictive ability of preoperative CAR for the defined endpoints was evaluated using receiver operating characteristic (ROC) analysis. The area under the curve (AUC) and Youden Index (YI) were used to determine an optimal CAR cut-off for the prediction of 3-, 5-, and 10-year RS and GS. The model goodness-of-fit was assessed using the Hosmer–Lemeshow chi^2^ test. Group comparisons were conducted using the Mann–Whitney U test or the Chi-square test. Survival analyses were carried out using the Kaplan–Meier method and the log-rank test. Independent predictive factors for RS and GS were identified using uni- and multivariable Cox regression. Multicollinearity was assessed using variance inflation factors (VIF). All *p*-values < 0.05 were considered statistically significant. Statistical analysis was performed using SPSS Statistics v29 (IBM Corp., Armonk, NY, USA), and graphs were generated using Prism v9 (GraphPad Software, La Jolla, CA, USA).

## 3. Results

A total of 532 consecutive liver transplantations were carried out within the study period. After excluding living-donor (n = 12), split-/domino transplantations (n = 4), re-transplantation within our institution (n = 43), patients dying within 90 days after transplantation (n = 47), as well as patients with EAD (n = 114), 330 patients were eligible for inclusion in this study.

### 3.1. ROC and Group Analysis

The ROC analysis showed a good model fit and AUC values: AUC = 0.70 for 3-year RS and AUC = 0.71 for 3-year GS. For 5-year RS and GS, AUC values were 0.65 and 0.66, respectively (Supplementary Table S1). Most patients were male (n = 176, 53%). The median donor and recipient ages were 57 (49–70) and 54 (48–63) years, respectively. The median labMELD was 16 (10–26), and 40 (12%) patients required renal replacement therapy preoperatively. The most common indication for transplantation was hepatocellular carcinoma (HCC, 29%), followed by alcoholic liver cirrhosis (19%) and primary sclerosing cholangitis (PSC) or primary biliary cirrhosis (PBC, combined 12%).

Cerebrovascular accident (CVA) was the most common cause of donor death, accounting for 55% of cases, followed by anoxia (25%) and trauma (12%). Donor and recipient characteristics are summarized in Table 1. Prior to transplantation, 122 patients (37%) were hospitalized, and 56 (17%) required intensive care unit (ICU) treatment. The median cold ischemia time was 510 (432–583) minutes, while the warm ischemia time was 47 (40–52) minutes. Intraoperatively, transfused blood products comprised mainly fresh frozen plasma (FFP), with a median of 19 (12–23) units, followed by red blood cells (RBC) with a median of 8 (3–10) units. Within the first 12 months post-transplantation, 21 patients (8%) died. At 120 months, 249 (76%) patients were still alive. A detailed summary of patient and donor characteristics, as well as perioperative outcomes, is provided in Table 1 and Table 2.

Using YI analysis, an ideal cut-off value of CAR = 41% (YI = 0.35) was defined for all endpoints (Supplementary Table S1). Applying this, we divided the study population into two groups (CAR ≤ 41% and CAR > 41%), with median CAR values of 10% (4–22%) and 101% [68–161%], respectively. The group with CAR > 41% (n = 142) showed a significantly higher proportion of patients with acute liver failure (ALF, 15% vs. 6%, *p* = 0.007), while the opposite was true for HCC patients (18% vs. 36%, *p* < 0.001). The median labMELD (23 vs. 11, *p* < 0.001), BAR (10 vs. 5, *p* < 0.001), and SOFT (15 vs. 8, *p* < 0.001) scores were significantly higher in the CAR > 41% group, as were the need for preoperative ICU stay (34% vs. 4%, *p* < 0.001) and the rate of renal replacement therapy (RRT, 18% vs. 8%, *p* < 0.001) before transplantation. Demographic and perioperative outcomes of the two groups are detailed in Table 1 and Table 2.

### 3.2. Association of Preoperative CAR with Long-Term Outcomes

The patients included in this study had a mean RS of 112 months (95%CI 106–119 months) and a mean GS of 110 months (95%CI 103–116 months). The median follow-up was 78 months for RS (95%CI 71.65–84.35) as well as for GS (95%CI 71.67–84.33). Only one recipient was lost to follow-up during the observation period. The group with CAR > 41% had a significantly shorter mean RS (98 months vs. 124 months, *p* < 0.001) and GS (93 months vs. 122 months, *p* < 0.001). Median RS and GS for the CAR > 41% group were 111 months (95%CI 93–119 months) and 106 months (95%CI 85–121 months), respectively. Median RS and GS could not be calculated for patients with CAR ≤ 41%, as not enough events occurred during the study period.

**Figure 1:**
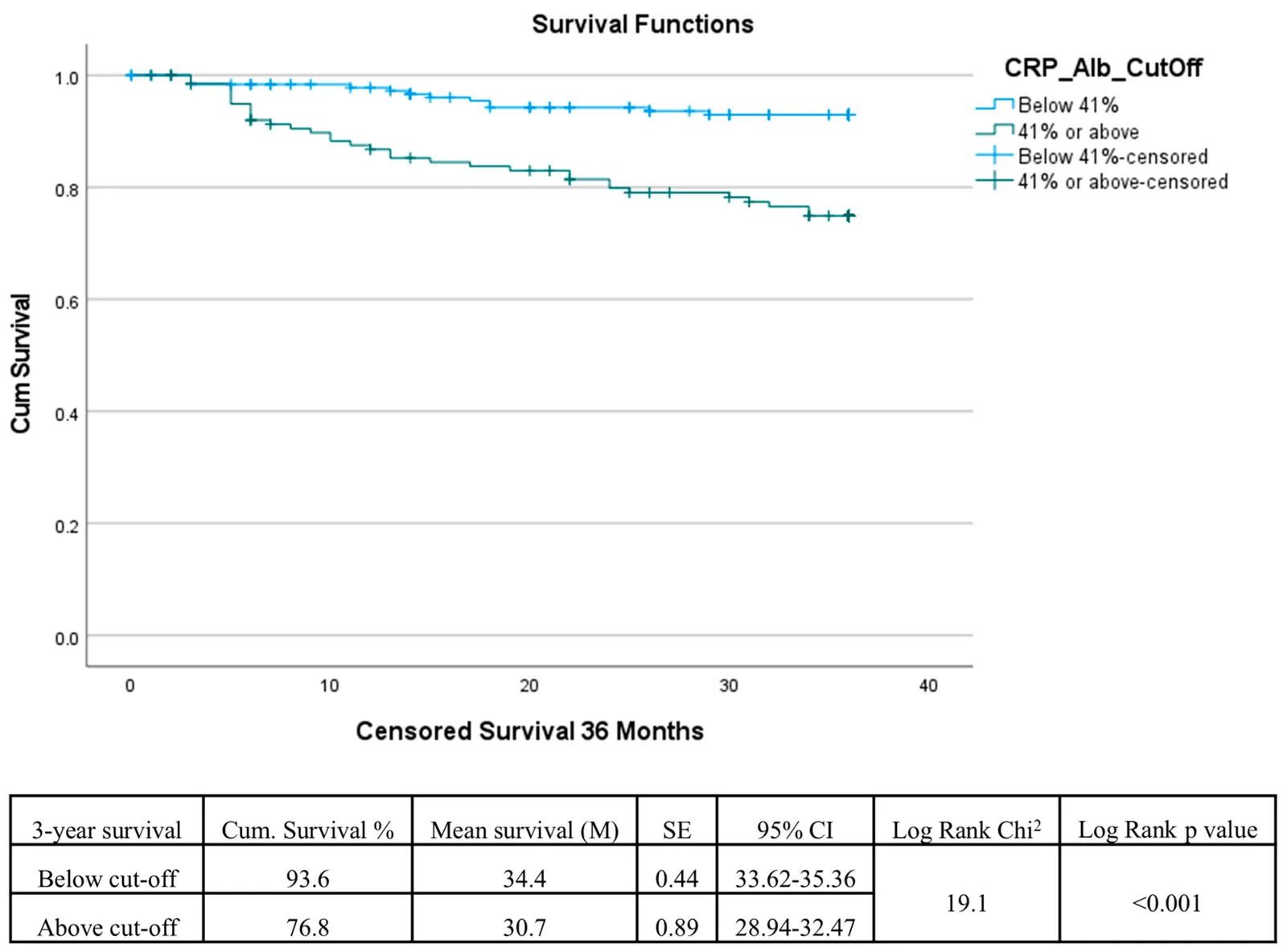
Comparison of 3-year recipient survival between patients with CAR ≤ 41% and CAR > 41%.

**Figure 2:**
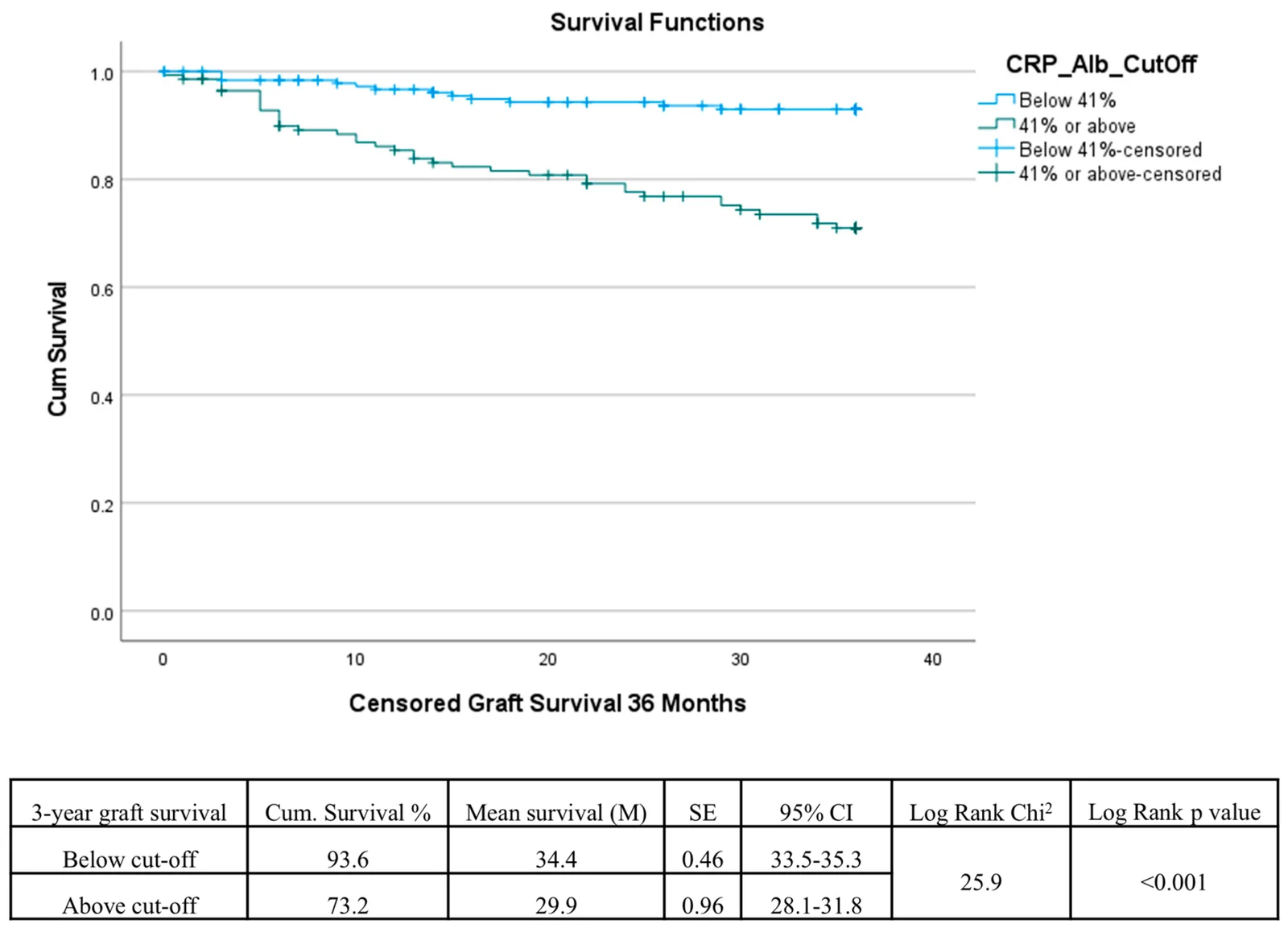
Comparison of 3-year graft survival between patients with CAR ≤ 41% and CAR > 41%.

The best predictive ability of CAR was shown for 3-year RS (AUC = 0.70, *p* < 0.001) and 3-year GS (AUC = 0.71, *p* < 0.001). As seen in Figure 1 and Figure 2, patients with CAR > 41% had significantly lower mean 3-year RS (31 vs. 34 months, *p* < 0.001) and 3-year GS (30 vs. 34 months, *p* < 0.001). Forty-six (13.9%) recipients died by 36 months and 57 (17.3%) within 5 years. Graft-loss events occurred in 51 (15.5%) cases within 3 years and 64 (19.4%) within 5 years.

Cox regression analysis identified CAR > 41% as the only independent predictor for both 3-year RS (HR 2.10, 95%CI 1.277–3.456, *p* = 0.003) and 3-year GS (HR 3.67, 95%CI 1.804–7.449, *p* < 0.001). Potential multicollinearity among MELD, BAR, and SOFT was assessed using variance inflation factor (VIF) and tolerance analyses. The VIF values were 2.595 for MELD, 2.082 for BAR, and 6.229 for SOFT, with corresponding tolerance values of 0.385, 0.480, and 0.161, respectively, indicating relevant collinearity, particularly for SOFT. We therefore selected BAR as the prognostic score for the multivariable model, as it already incorporates MELD, and included it together with CAR and other predefined covariates. Detailed results are outlined in Table 3 and Table 4.

Additional separate multivariable Cox regression models incorporating MELD and SOFT instead of BAR were also performed to assess the robustness of the findings and are presented in the Supplementary Material (Supplementary Tables S8–S23).

Similar results were obtained from Cox regression analysis for 5-year RS and GS. Here, CAR ≥ 41% was an independent predictor of 5-year RS (HR 2.097, 95%CI 1.270–3.463, *p* = 0.004) and GS (HR 2.842, 95%CI 1.576–5.126, *p* ≤ 0.001).

Further details on 5-year endpoints can be found in Supplementary Tables S2 and S3 and Supplementary Figures S1 and S2.

### 3.3. Association of Preoperative CAR with Long-Term Outcomes in Patients with EAD and 90-Days Mortality

A sensitivity analysis was performed including patients with EAD and patients who died within 90 days post-transplant. A total of 478 patients were selected. In the Cox regression analysis CAR ≥ 41% remained an independent predictor for 3-year RS (HR 2.025, 95%CI 1.407–2.913, *p* ≤ 0.001) and GS (2.242, 95%CI 1.442–3.487, *p* ≤ 0.001). Moreover, postoperative FFP units were also significant for 3-year RS (HR 1.029, 95%CI 1.008–1.050, *p* = 0.006) and GS (HR 1.031, 95%CI 1.008–1.055, *p* = 0.009). Similar results for CAR ≥ 41% were obtained for 5-year RS (HR 2.040, 95%CI 1.415–2.942, *p* ≤ 0.001) and GS (HR 2.180, 95%CI 1.470–3.234, *p* ≤ 0.001). Detailed results are given in Supplementary Tables S4–S7.

## 4. Discussion

This study demonstrated that a higher CAR is significantly associated with increased long-term graft loss and recipient mortality following DDLT in patients without EAD. Patients with CAR > 41% had a more than threefold increased risk of graft loss within three years, with similar trends observed for five-year graft loss and overall mortality. These findings suggest that CAR may provide added prognostic value for long-term outcomes post-transplant, especially in patients not suffering from early postoperative death or EAD.

The MELD score has long served as an effective predictor of mortality risk in patients awaiting liver transplantation, guiding allocation and prioritization. However, its prognostic utility after transplantation—particularly over the long term—remains limited. Bleszynski et al. reported no association between pre-transplant MELD and long-term survival, a finding mirrored in our cohort. Similarly, while the BAR and SOFT scores have demonstrated predictive ability for short-term (3–12 month) outcomes in some studies [1,9,20,21], neither proved independently predictive in our multivariable model. This may be due in part to our exclusion of patients who died within 90 days post-transplant, which could diminish the short-term predictive advantage of these scores.

The predictive value of CAR may be attributable to its components: CRP, a marker of systemic inflammation, and albumin, a surrogate for nutritional and physiological reserve. Both are synthesized in the liver and reflect the patient’s overall health status. Elevated CRP levels signal heightened inflammatory activity, which may stem from underlying malignancy, infection, or cirrhosis [22,23,24]. Low albumin levels are commonly associated with poor nutritional status and have been linked to adverse post-operative outcomes [25,26]. The combined index, CAR, has previously shown prognostic utility in various clinical settings, including patients with malignancies [13,27], critically ill patients [28], and those undergoing major abdominal surgeries, including liver resections [29,30] and transplantation [16,31,32].

This study builds on our previous work [16], where we reported an association between CAR and short-term outcomes following DDLT. Among 390 patients, a cut-off of 26% demonstrated a higher post-liver-transplant morbidity, with a higher and more severe complication rate within the first 90 days. It also indicated a higher mortality rate for the first year after transplantation [16]. More recently, Kim et al. showed that CAR was also linked to 1-year mortality [31]. They included a larger population (n = 3614) and calculated the best cut-off at 34%. They showed a 1.4-fold higher risk of 1-year mortality for CAR ≥ 34% and were able to confirm that CAR is an independent predictor of 1-year mortality in DDLT recipients.

Predictive effects have also been demonstrated in living-donor liver transplantation (LDLT). Here, Park et al. found that higher CAR levels were consistently associated with an increased incidence of early EAD in 588 LDLT recipients [33]. Specifically, a CAR of >20% was associated with a 2-fold higher risk of EAD and was an independent risk factor in multivariable analysis [33]. In kidney transplantation, Kwon et al. identified increased CAR as a risk factor for worse survival outcomes. After dividing their study population into quartiles based on CAR levels, they were able to show that patients in the top quartile had the lowest long-term survival of 15 years [32]. However, their median CAR value was 2.5%, which is much lower than in studies on liver transplantation or resection [16,31,34], or other surgical–oncological cohorts [35,36].

To our knowledge, this is the first study demonstrating a clear predictive relationship between preoperative CAR and long-term RS and GS after DDLT. Interestingly, in contrast to our previous work and Kim et al., CAR in this study remained independently associated with long-term recipient and graft survival in multivariable analyses and was the only independent predictor in multivariable analyses [16,31]. However, both our previous and current studies have failed to define the interplay between CAR and EAD. In our first paper, no clear association was found between the two, with both groups of patients exhibiting similar rates of EAD [16]. In this study, patients with EAD were excluded, as the negative influence of EAD on RS and GS has already been established [5]. These patients are at higher risk of adverse outcomes, regardless of CAR [5,16]. Further studies are necessary to elucidate the relationship between CAR, EAD and their effect on long-term outcomes. In our previous study, we defined a CAR of 26% as the threshold for increased risk of perioperative morbidity and mortality [16]. Here, the cut-off value for increased long-term mortality and graft loss was defined as 41%. Hence, patients could theoretically be stratified according to these two cut-offs into three groups with increasing risk of adverse outcomes. In the first (CAR < 26%), patients would be at normal or reduced risk of post-DDLT morbidity or mortality. In the second (CAR 26–41%), there would be increased risk of perioperative adverse events, but not long-term mortality or graft loss. Finally, the group with CAR > 41% would face the highest risk, with survivors of short-term complications still being in danger of long-term graft loss and mortality. Of course, further validation in external cohorts and prospective evaluation is necessary to establish the clinical usefulness and transferability of such a stratification.

Our findings must be interpreted in light of several limitations. This was a retrospective, single-center study, limiting generalizability. The primary analysis was restricted to recipients without EAD and without mortality within 90 days after transplantation, as our aim was to evaluate the association between preoperative CAR and long-term outcomes beyond the early post-transplant period. However, this approach may introduce selection bias and limit the generalizability of our findings. In addition, EAD may potentially represent an intermediate pathway between elevated preoperative CAR and subsequent mortality, and its exclusion could therefore attenuate part of the association under investigation. To address this concern, we performed a sensitivity analysis including the full cohort of 478 recipients, including patients with EAD and those who died within 90 days after transplantation. The persistence of the association between preoperative CAR and long-term recipient survival in this full-cohort analysis supports the robustness of our findings. Nevertheless, our study cannot determine whether EAD mediates the association between preoperative CAR and long-term outcomes. Although we adjusted for various confounding factors through multivariable analysis, residual confounding cannot be entirely excluded. Moreover, the study focused on all-cause mortality and graft loss, without stratifying by specific causes (e.g., infection, rejection, or malignancy recurrence). This limits our ability to determine whether CAR is more predictive in certain etiologies of graft failure or patient death. Additionally, CAR was assessed at a single preoperative time point, providing only a static view of the patient’s condition. Further prospective studies are needed to investigate the role of dynamic CAR trends over time and their potential correlation with long-term outcomes. Importantly, while CAR reflects the recipient’s systemic condition, it does not account for donor quality or technical factors involved in the transplant procedure. Furthermore, CAR has previously been reported to provide greater prognostic accuracy than its individual components, CRP and albumin [12,29,30], it remains an indirect marker of systemic inflammation and nutritional status. Concomitant inflammatory conditions may influence CAR values and thus affect its interpretation, particularly when assessing long-term transplant outcomes. Furthermore, longitudinal changes in inflammatory and nutritional status were not evaluated. CAR was assessed preoperatively and therefore reflects the inflammatory and nutritional status at a single time point before transplantation. Previous studies have demonstrated that postoperative changes in inflammatory markers may provide additional prognostic information after liver transplantation. In a prospective single-center cohort study, Seller-Pérez et al. evaluated CRP measurements after liver transplantation and found that a blunted postoperative CRP increase was associated with poor allograft function and hospital mortality [37]. Consequently, our study cannot determine whether postoperative changes or trajectories of CAR, CRP, or albumin are associated with long-term recipient or graft outcomes. Future studies incorporating serial measurements may provide further insight into the prognostic relevance of dynamic changes in inflammatory and nutritional status after transplantation.

Despite these challenges, CAR is a simple, blood-laboratory-derived metric that may offer valuable additional insight into long-term prognosis following liver transplantation. While it should not replace comprehensive clinical evaluation by experienced transplant teams, CAR could serve as a useful adjunct for risk stratification, with relevance for identifying patients at increased risk of graft failure and mortality. Further validation in large, multi-centric prospective cohorts is warranted to confirm these findings and determine how best to integrate CAR into clinical decision-making.

## Figures and Tables

**Table 1:** Donor and recipient characteristics for patients with a preoperative CAR below and above the cut-off value of 41%.

Variables	All Patients (n = 330)	CAR ≤ 41% (n = 188)	CAR > 41% (n = 142)	*p*-Value
**Donor age (years)**	57 (49–70)	61 (50–71)	57 (47–67)	0.150
**Donor BMI**	28 (24–29)	28 (25–31)	26 (24–29)	0.172
**Donor sex ratio (F/M)**	154 (47%)/176 (53%)	88 (47%)/100 (53%)	66 (47%)/76 (53%)	0.521
**Cause of donor death**	CVA 182 (55%) Anoxia 81 (25%) Trauma 41 (12%) Other 26 (8%)	CVA 91 (48%) Anoxia 54 (29%) Trauma 27 (14%) Other 16 (9%)	CVA 91 (64%) Anoxia 27 (19%) Trauma 14 (10%) Other 10 (7%)	0.005 0.052 0.242 0.684
**Allocation type**	Local 20 (6%) Regional 165 (50%) National 145 (44%)	Local 14 (7%) Regional 97 (52%) National 78 (41%)	Local 6 (5%) Regional 68 (48%) National 67 (47%)	0.373 0.824 0.309
**Recipient age (years)**	54 (48–63)	54 (49–63)	58 (48–63)	0.713
**Recipient BMI**	28 (23–30)	29 (23–30)	26 (23–30)	0.345
**Recipient sex ratio (F/M)**	101 (31%)/229 (69%)	54 (29%)/134 (71%)	47 (33%)/95 (67%)	0.401
**Etiology of liver disease**	ALF 33 (10%) HCC 96 (29%) Alc. cirrhosis 63 (19%) Viral 27 (8%) PSC/PBC 39 (12%) AIH 5 (2%) Graft failure 2 (1%) Other 65 (19%)	ALF 11 (6%) HCC 69 (36%) Alc. cirrhosis 31 (16%) Viral 17 (9%) PSC/PBC 17 (9%) AIH 2 (2%) Graft failure 0 (0%) Other 41 (22%)	ALF 22 (15%) HCC 27 (18%) Alc. cirrhosis 32 (23%) Viral 10 (7%) PSC/PBC 22 (16%) AIH 3 (2%) Graft failure 2 (1%) Other 24 (18%)	0.008 <0.001 0.209 0.550 0.085 0.655 0.091 0.578
**labMELD**	16 (10–26)	11 (8–19)	23 (16–32)	<0.001
**BAR Score**	8 (3–12)	5 (3–9)	10 (7–14)	<0.001
**SOFT Score**	11 (7–16)	8 (5–13)	15 (10–21)	<0.001
**Recipient pre-OLT ICU stay**	56 (17%)	8 (4%)	48 (34%)	<0.001
**Recipient inpatient pre-OLT**	122 (37%)	39 (21%)	83 (59%)	<0.001
**Recipient pre-OLT** **abdominal surgery**	95 (29%)	55 (29%)	40 (28%)	0.902
**Recipient pre-OLT** **encephalopathy**	124 (38%)	57 (30%)	67 (47%)	0.002
**Recipient pre-OLT ascites**	181 (55%)	73 (39%)	108 (76%)	<0.001
**Pre-OLT renal failure**	90 (27%)	35 (19%)	55 (39%)	<0.001
**Pre-OLT RRT**	40 (12%)	15 (8%)	25 (18%)	0.010
**Pre-OLT CRP (mg/L)**	9.5 (3.0–26.0)	3.8 (1.4–7.2)	28.6 (19.2–44.9)	<0.001
**Pre-OLT Alb (g/dL)**	3.3 (2.8–3.9)	3.7 (3.2–4.2)	2.9 (2.5–3.3)	<0.001
**Pre-OLT CAR (%)**	31.5 (9.0–87.5)	10.4 (3.9–22.3)	101.3 (67.9–160.5)	<0.001

Values given as median [interquartile range Q1–Q3], standard deviation or absolute and relative frequencies; abbreviations used: CAR, C-reactive protein-to-albumin ratio; CRP, C-reactive protein; Alb, albumin; BMI, body mass index; CVA, cerebrovascular accident; OLT, orthotopic liver transplantation; ALF, acute liver failure; HCC, hepatocellular carcinoma; Alc., alcoholic; PSC, primary sclerosing cholangitis; PBC, primary biliary cirrhosis; AIH, autoimmune hepatitis; labMELD, laboratory model for end-stage liver disease; BAR, balance of risk; SOFT, survival outcomes following liver transplantation; ICU, intensive care unit; RRT, renal replacement therapy.

**Table 2:** Perioperative outcomes for patients with a preoperative CAR below and above the cut-off value of 41%.

Variables	All Patients (n = 330)	CAR ≤ 41% (n = 188)	CAR > 41% (n = 142)	*p*-Value
**Cold ischemia time** **(minutes)**	510 (432–583)	498 (425–572)	525 (437–589)	0.639
**Warm ischemia time** **(minutes)**	47 (40–52)	46 (41–52)	46 (40–51)	0.937
**Intraoperative RBC units**	8 (3–10)	7 (3–10)	9 (5–12)	0.036
**Intraoperative FFP units**	19 (12–23)	19 (12–23)	18 (12–21)	0.729
**Postoperative RBC units ^a^**	2 (0–3)	1 (0–2)	3 (0–4)	0.086
**Postoperative FFP units ^a^**	3 (0–4)	2 (0–4)	3 (0–4)	0.524

Values given as median [interquartile range Q1–Q3], standard deviation or absolute and relative frequencies; Abbreviations used: CAR, C-reactive protein-to-albumin ratio; RBC, red blood cell; FFP, fresh frozen plasma. ^a^ Refers to blood products given during the first 7 days following OLT. For RS and GS mostly, no median was given; values were then given as mean months [CI interval].

**Table 3:** Association of perioperative factors with 3-year recipient survival.

Perioperative Factor	Univariable Cox Regression Analysis		Multivariable Cox Regression Analysis	
	**HR (95%CI)**	***p*** **-Value**	**HR (95%CI)**	***p*** **-Value**
**CAR > 41%**	3.902 (2.015–7.555)	<0.001	2.101 (1.277–3.456)	0.003
**Donor age (years)**	0.998 (0.980–1.016)	0.828		
**Donor BMI**	0.968 (0.918–1.020)	0.193		
**Donor sex (male)**	1.027 (0.572–1.844)	0.930		
**Cause of donor death**	1.020 (0.749–1.391)	0.899		
**Allocation type**	1.209 (0.721–2.026)	0.468		
**Recipient age**	1.020 (0.990–1.050)	0.181		
**Recipient BMI**	0.998 (0.977–1.019)	0.840		
**Recipient sex (male)**	0.533 (0.296–0.960)	0.040		
**Etiology of liver disease**	1.004 (0.887–1.136)	0.950		
**labMELD**	1.047 (1.020–1.075)	<0.001		
**BAR score**	1.087 (1.028–1.148)	0.003	0.995 (0.942–1.051)	0.861
**SOFT score**	1.046 (1.014–1.080)	0.004		
**Recipient pre-OLT ICU (yes)**	3.018 (1.639–5.559)	<0.001		
**Recipient pre-OLT** **abdominal surgery (yes)**	0.662 (0.328–1.338)	0.234		
**Recipient inpatient pre-OLT (yes)**	2.445 (1.357–4.403)	0.003	1.116 (0.635–1.961)	0.703
**Recipient pre-OLT** **encephalopathy (yes)**	1.855 (1.034–3.328)	0.039		
**Recipient pre-OLT ascites (yes)**	1.207 (0.665–2.191)	0.535		
**Pre-OLT renal failure (yes)**	2.291 (1.272–4.126)	0.006	1.364 (0.817–2.278)	0.236
**Pre-OLT RRT (yes)**	2.618 (1.326–5.169)	0.011		
**Cold ischemia time (minutes)**	1.000 (0.997–1.002)	0.967		
**Warm ischemia time (minutes)**	1.024 (0.994–1.056)	0.129		
**Intraoperative RBC units**	1.047 (1.022–1.073)	0.002		
**Intraoperative FFP units**	1.014 (0.987–1.041)	0.314		
**Postoperative RBC units ^a^**	1.088 (1.042–1.137)	<0.001	1.034 (0.977–1.094)	0.247
**Postoperative FFP units ^a^**	1.067 (1.019–1.117)	0.005	1.030 (0.984–1.077)	0.204

Results given as hazard ratios (HR) with 95% confidence intervals (95%CI). Factors showing significant results in the univariable analysis were included in the multivariable Cox regression model. To avoid multicollinearity, certain variables found to be significantly different in the subgroup analysis were not included in the multivariable Cox regression analysis (e.g., postoperative RBC and FFP, etc.). Abbreviations used: CAR, C-reactive protein-to-albumin ratio; CRP, C-reactive protein; Alb, albumin; BMI, body mass index; labMELD, laboratory model of end-stage liver disease score; BAR, balance of risk; SOFT, survival outcomes following liver transplantation; OLT, orthotopic liver transplantation; ICU, intensive care unit; RRT, renal replacement therapy; RBC, red blood cell; FFP, fresh frozen plasma. ^a^ Refers to blood products given during the first 7 days following OLT.

**Table 4:** Association of perioperative factors with 3-year graft survival.

Perioperative Factor	Univariable Cox Regression Analysis		Multivariable Cox Regression Analysis	
	**HR (95%CI)**	***p*** **-Value**	**HR (95%CI)**	***p*** **-Value**
**CAR > 41%**	4.605 (2.406–8.813)	<0.001	3.666 (1.804–7.449)	<0.001
**Donor age (years)**	1.001 (0.984–1.018)	0.918		
**Donor BMI**	0.987 (0.944–1.031)	0.536		
**Donor sex (male)**	0.880 (0.505–1.531)	0.650		
**Cause of donor death**	0.928 (0.682–1.262)	0.633		
**Allocation type**	1.048 (0.647–1.697)	0.848		
**Recipient age**	1.014 (0.987–1.042)	0.305		
**Recipient BMI**	0.995 (0.968–1.023)	0.671		
**Recipient sex (male)**	0.589 (0.336–1.033)	0.070		
**Etiology of liver disease**	1.008 (0.898–1.133)	0.888		
**labMELD**	1.045 (1.019–1.071)	<0.001		
**BAR score ^a^**	1.074 (1.019–1.132)	0.009	1.006 (0.941–1.076)	0.853
**SOFT score**	1.043 (1.012–1.074)	0.010		
**Recipient pre-OLT ICU (yes)**	2.872 (1.599–5.158)	<0.001		
**Recipient pre-OLT** **abdominal surgery (yes)**	0.646 (0.331–1.262)	0.183		
**Recipient inpatient pre-OLT (yes)**	2.545 (1.455–4.451)	0.001	1.494 (0.744–3.000)	0.259
**Recipient pre-OLT** **encephalopathy (yes)**	1.496 (0.858–2.610)	0.159		
**Recipient pre-OLT ascites (yes)**	1.461 (0.820–2.603)	0.191		
**Pre-OLT renal failure (yes)**	1.869 (1.061–3.292)	0.035	0.968 (0.505–1.855)	0.923
**Pre-OLT RRT (yes)**	2.238 (1.146–4.372)	0.029		
**Cold ischemia time (minutes)**	0.999 (0.997–1.002)	0.633		
**Warm ischemia time (minutes)**	1.022 (0.993–1.052)	0.148		
**Intraoperative RBC units**	1.043 (1.018–1.070)	0.005		
**Intraoperative FFP units**	1.004 (0.976–1.033)	0.776		
**Postoperative RBC units ^a^**	1.082 (1.036–1.129)	0.003	1.067 (0.998–1.135)	0.062
**Postoperative FFP units ^a^**	1.067 (1.022–1.114)	0.008	1.018 (0.963–1.076)	0.532

Results given as hazard ratios (HR) with 95% confidence intervals (95%CI). Factors showing significant results in the univariable analysis were included in the multivariable Cox regression model. Abbreviations used: CAR, C-reactive protein-to-albumin ratio; CRP, C-reactive protein; Alb, albumin; BMI, body mass index; labMELD, laboratory model of end-stage liver disease score; BAR, balance of risk; SOFT, survival outcomes following liver transplantation; OLT, orthotopic liver transplantation; ICU, intensive care unit; RRT, renal replacement therapy; RBC, red blood cell; FFP, fresh frozen plasma. ^a^ Refers to blood products given during the first 7 days following OLT.

## Data Availability

Data used and generated during this study can be made available upon reasonable request to the corresponding author.

## Supplementary Materials

The following supporting information can be downloaded at: https://www.mdpi.com/article/10.3390/jcm15187063/s1, Figure S1: Comparison of 5-year recipient survival between patients with CAR ≤ 41% and CAR > 41%, Figure S2: Comparison of 5-year graft survival between patients with CAR ≤ 41% and CAR > 41%, Table S1: ROC analysis for different endpoints, Table S2: Association of perioperative factors with 5-year recipient survival, Table S3: Association of perioperative factors with 5-year graft survival, Table S4: Association of perioperative factors with 3-year recipient survival in patients with EAD and 90-day mortality, Table S5: Association of perioperative factors with 3-year graft survival in patients with EAD and 90-day mortality, Table S6: Association of perioperative factors with 5-year recipient survival in patients with EAD and 90-day mortality, Table S7: Association of perioperative factors with 5-year graft survival in patients with EAD and 90-day mortality, Table S8: Association of perioperative factors with 3-year recipient survival (incl. MELD score multivariable analysis), Table S9: Association of perioperative factors with 3-year graft survival (incl. MELD score multivariable analysis), Table S10: Association of perioperative factors with 5-year recipient survival (incl. MELD score multivariable analysis), Table S11: Association of perioperative factors with 5-year graft survival (incl. MELD score multivariable analysis), Table S12: Association of perioperative factors with 3-year recipient survival (incl. SOFT score multivariable analysis), Table S13: Association of perioperative factors with 3-year graft survival (incl. SOFT score multivariable analysis), Table S14: Association of perioperative factors with 5-year recipient survival (incl. SOFT score multivariable analysis), Table S15: Association of perioperative factors with 5-year graft survival (incl. SOFT score multivariable analysis), Table S16: Association of perioperative factors with 3-year recipient survival in patients with EAD and 90-day mortality (incl. MELD score multivariable analysis), Table S17: Association of perioperative factors with 3-year graft survival in patients with EAD and 90-day mortality (incl. MELD score multivariable analysis), Table S18: Association of perioperative factors with 5-year recipient survival in patients with EAD and 90-day mortality (incl. MELD score multivariable analysis), Table S19: Association of perioperative factors with 5-year graft survival in patients with EAD and 90-day mortality (incl. MELD score multivariable analysis), Table S20: Association of perioperative factors with 3-year recipient survival in patients with EAD and 90-day mortality (incl. SOFT score multivariable analysis), Table S21: Association of perioperative factors with 3-year graft survival in patients with EAD and 90-day mortality (incl. SOFT score multivariable analysis), Table S22: Association of perioperative factors with 5-year recipient survival in patients with EAD and 90-day mortality (incl. SOFT score multivariable analysis), Table S23: Association of perioperative factors with 5-year graft survival in patients with EAD and 90-day mortality (incl. SOFT score multivariable analysis).

## Abbreviations

The following abbreviations are used in this manuscript:

Alb	Albumin
AUC	area under the curve
BAR	Balance of Risk
BMI	body mass index
CAR	C-reactive protein-to-albumin ratio
CRP	C-reactive protein
CVA	Cerebrovascular accident
DDLT	deceased-donor liver transplantation
EAD	early allograft dysfunction
FFP	fresh frozen plasma
GS	graft survival
HCC	hepatocellular carcinoma
ICU	intensive care unit
MELD	Model for End-Stage Liver Disease
OLT	orthotopic liver transplantation
PBC	primary biliary cirrhosis
PSC	primary sclerosing cholangitis
RBC	red blood cells
ROC	receiver operating characteristic
RRT	renal replacement therapy
RS	recipient survival
SOFT	Survival Outcomes Following Liver Transplantation
YI	Youden Index
