# Supplementary material for: Preoperative C-Reactive Protein-to-Albumin Ratio Predicts Recipient and Graft Survival After Deceased-Donor Liver Transplantation
Source: J Clin Med. 2026 Sep 11;15(18):7063. doi: 10.3390/jcm15187063 (PMC13607958; doi:10.3390/jcm15187063)
Supplement: Supplementary file 1 [file jcm-15-07063-s001.zip › jcm-4450189-supplementary.pdf]

Supplementary Material

Preoperative C-Reactive Protein-to-Albumin Ratio Predicts Recipient and Graft Survival After Deceased-Donor Liver Transplantation

David Erren [1], Franziska A. Meister [1], Karsten Große [2], Tony Bruns [2], Naomi Gestels [3], Oliver Beetz [1], Felix Oldhafer [1], Martin W. von Websky [1], Thomas Vogel [1], Florian W. R. Vondran [1] and Iakovos Amygdalos [1,*]

[1] Department of General, Visceral, Pediatric, and Transplantation Surgery, University Hospital RWTH Aachen, Pauwelsstraße 30, 52074 Aachen, Germany
[2] Department of Internal Medicine III, University Hospital RWTH, Pauwelsstraße 30, 52074 Aachen, Germany
[3] Department of Internal Medicine, Maastricht University Medical Centre, 6229 HX Maastricht, The Netherlands
* Correspondence: iamygdalos@ukaachen.de

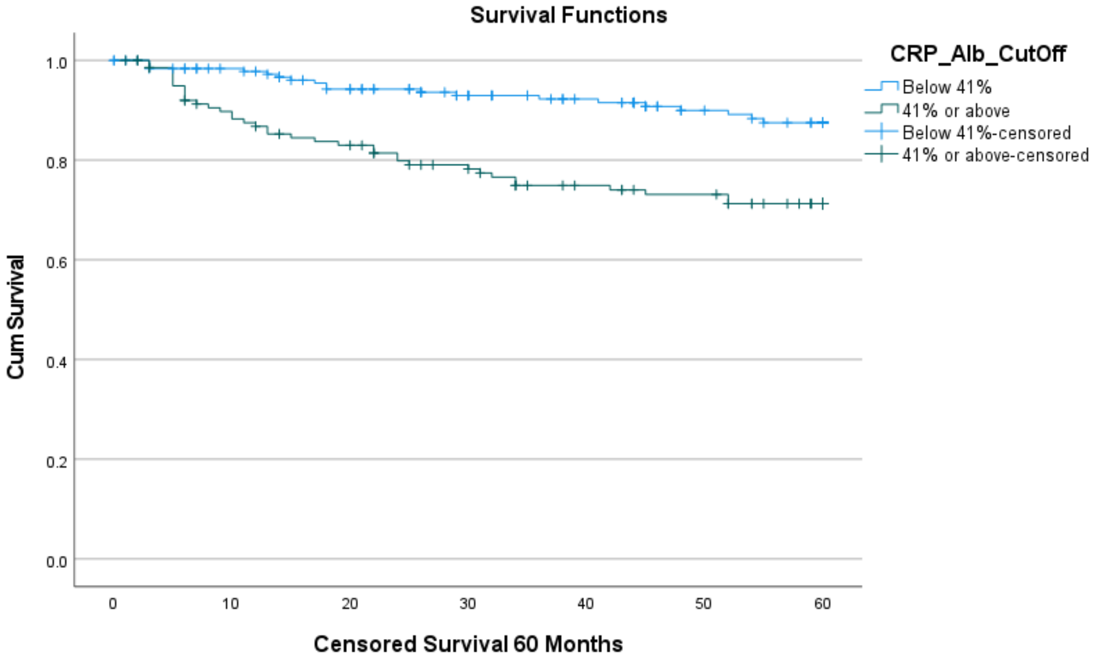

5-year survival	Cum. survival %	Mean survival (M)	SE	95% CI	Log-rank Chi²	Log-rank p-value
Below cut-off	89.9	56.1	0.93	54.3-57.9	14.3	<0.001
Above cut-off	73.9	48.2	1.74	44.8-51.6		

Supplementary Figure S1. Comparison of 5-year recipient survival between patients with CAR≤41% and CAR>41%.

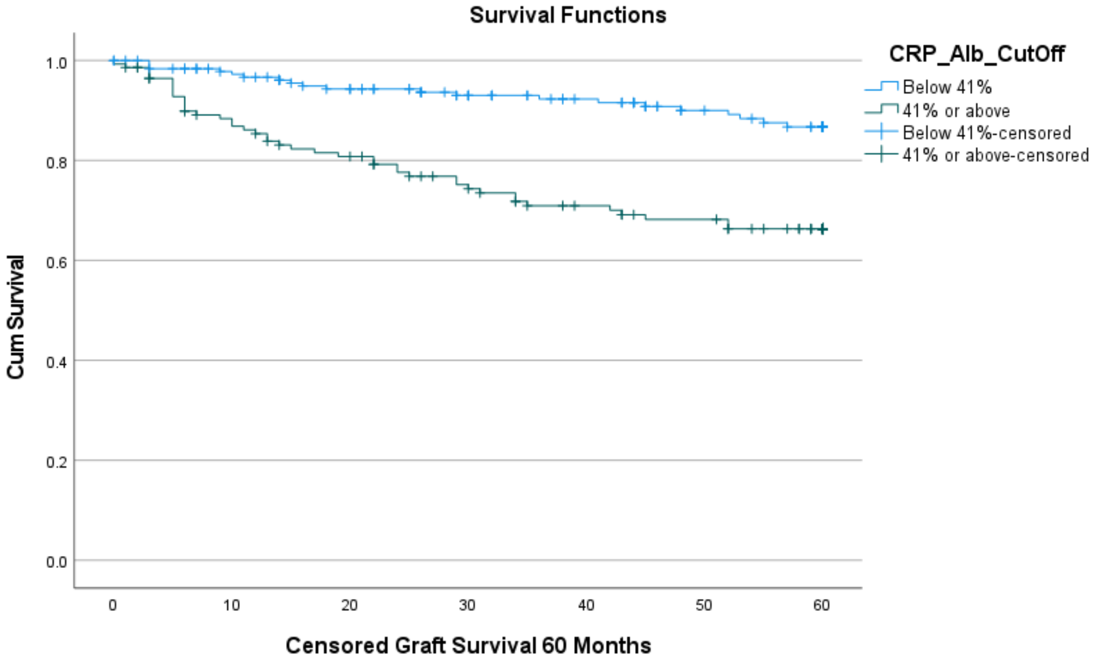

5-year graft survival	Cum. survival %	Mean survival (M)	SE	95% CI	Log-rank Chi²	Log-rank p-value
Below cut-off	89.4	56.0	0.93	54.2-57.8	20.5	<0.001
Above cut-off	69.7	46.3	1.83	42.7-49.9		

Supplementary Figure S2. Comparison of 5-year graft survival between patients with CAR≤41% and CAR>41%.

Supplementary Table S1. ROC analysis for different endpoints

Endpoint	AUC	SE	95% CI	*p*	Positive if ≥ to (%)	Sensitivity	Specificity	Youden	HL Chi^2	*p*
**36M-RS**	0.700	0.041	0.620-0.780	<0.001	41.125	0.733	0.379	0.354	12.061	0.149
**36M-GS**	0.712	0.038	0.637-0.786	<0.001	41.125	0.760	0.368	0.392	14.391	0.072
**60M-RS**	0.647	0.042	0.566-0.728	0.001	41.125	0.661	0.380	0.281	13.093	0.109
**60M-GS**	0.660	0.038	0.584-0.735	<0.001	41.125	0.683	0.367	0.315	13.376	0.100

Supplementary Table S2. Association of perioperative factors with 5-year recipient survival

**Perioperative factor**	**Univariable Cox regression analysis**		**Multivariable Cox regression analysis**	
	HR (95% CI)	*p*-value	HR (95% CI)	*p*-value
**CAR>41%**	2.775 (1.596-4.826)	<0.001	2.097 (1.270-3.463)	**0.004**
**Donor age (years)**	1.005 (0.989-1.022)	0.523		
**Donor BMI**	0.969 (0.926-1.015)	0.185		
**Donor sex (male)**	1.064 (0.629-1.799)	0.817		
**Cause of donor death**	1.140 (0.873-1.489)	0.334		
**Allocation type**	1.125 (0.715-1.772)	0.610		
**Recipient age**	1.012 (0.987-1.038)	0.360		
**Recipient BMI**	0.993 (0.963-1.025)	0.678		
**Recipient sex (male)**	0.617 (0.362-1.050)	0.075		
**Etiology of liver disease**	0.985 (0.880-1.102)	0.787		
**labMELD**	1.038 (1.013-1.063)	0.002		
**BAR score**[a]	1.064 (1.013-1.118)	0.014	1.003 (0.949-1.059)	0.922
**SOFT score**[b]	1.035 (1.004-1.066)	0.024		
**Recipient pre-OLT ICU (yes)**	2.361 (1.335-4.174)	0.003		
**Recipient pre-OLT abdominal surgery (yes)**	0.545 (0.282-1.054)	0.071		
**Recipient inpatient pre-OLT (yes)**	1.738 (1.028-2.940)	0.039	1.058 (0.604-1.856)	0.843
**Recipient pre-OLT encephalopathy (yes)**	1.426 (0.842-2.416)	0.187		
**Recipient pre-OLT ascites (yes)**	1.140 (0.669-1.941)	0.630		
**Pre-OLT renal failure (yes)**	1.720 (1.001-2.955)	0.050	1.275 (0.769-2.116)	0.347
**Pre-OLT RRT (yes)**	2.003 (1.036-3.874)	0.039		
**Cold ischaemia time (minutes)**	1.000 (0.998-1.002)	0.815		
**Warm ischaemia time (minutes)**	1.020 (0.991-1.050)	0.186		
**Intraoperative RBC units**	1.042 (1.016-1.068)	0.001		
**Intraoperative FFP units**	1.012 (0.986-1.037)	0.371		
**Postoperative RBC units**[d]	1.079 (1.035-1.124)	<0.001	1.035 (0.978-1.094)	0.232
**Postoperative FFP units**[d]	1.051 (1.007-1.098)	0.024	1.025 (0.981-1.072)	0.270

Results given as hazard ratios (HR) with 95% confidence intervals (95% CI). Factors showing significant results in the univariable analysis were included in the multivariable Cox regression model. Abbreviations used: *CAR*, C-reactive protein-to-albumin ratio; *CRP*, C-reactive protein; *Alb*, albumin; *BMI*, body mass index; *labMELD*, laboratory model of end-stage liver disease score; *BAR*, balance of risk; *SOFT*, survival outcomes following liver transplantation; *OLT*, orthotopic liver transplantation; *ICU*, intensive care unit; *RRT*, renal replacement therapy; *RBC*, red blood cell; *FFP*, fresh frozen plasma. [a] Refers to Schlegel et al. [b] Refers to Rana et al. [c] Refers to blood products given during the first 7 days following OLT.

Supplementary Table S3. Association of perioperative factors with 5-year graft survival

Perioperative factor	Univariable Cox analysis		Multivariable Cox regression analysis	
	HR (95% CI)	*p*-value	HR (95% CI)	*p*-value
**CAR>41%**	3.183 (1.872-5.412)	<0.001	2.842 (1.576-5.126)	**<0.001**
**Donor age (years)**	1.009 (0.994-1.026)	0.246		
**Donor BMI**	0.986 (0.949-1.025)	0.490		
**Donor sex (male)**	0.930 (0.568-1.525)	0.774		
**Cause of donor death**	1.022 (0.785-1.330)	0.874		
**Allocation type**	1.020 (0.668-1.557)	0.926		
**Recipient age**	1.006 (0.983-1.030)	0.601		
**Recipient BMI**	0.987 (0.941-1.034)	0.575		
**Recipient sex (male)**	0.657 (0.397-1.089)	0.103		
**Etiology of liver disease**	0.988 (0.889-1.097)	0.815		
**labMELD**	1.034 (1.011-1.058)	0.004		
**BAR score[a]**	1.049 (1.001-1.100)	0.045	0.995 (0.934-1.060)	0.881
**SOFT score[b]**	1.031 (1.003-1.060)	0.032		
**Recipient pre-OLT ICU (yes)**	2.214 (1.281-3.825)	0.004		
**Recipient pre-OLT abdominal surgery (yes)**	0.569 (0.309-1.048)	0.071		
**Recipient inpatient pre-OLT (yes)**	1.759 (1.071-2.888)	0.026	1.135 (0.610-2.114)	0.689
**Recipient pre-OLT encephalopathy (yes)**	1.146 (0.691-1.898)	0.598		
**Recipient pre-OLT ascites (yes)**	1.329 (0.799-2.210)	0.273		
**Pre-OLT renal failure (yes)**	1.395 (0.826-2.356)	0.213		
**Pre-OLT RRT (yes)**	1.693 (0.883-3.246)	0.113		
**Cold ischaemia time (minutes)**	1.000 (0.998-1.002)	0.763		
**Warm ischaemia time (minutes)**	1.016 (0.988-1.044)	0.266		
**Intraoperative RBC units**	1.037 (1.011-1.064)	0.005		
**Intraoperative FFP units**	1.002 (0.975-1.029)	0.902		
**Postoperative RBC units[d]**	1.070 (1.027-1.115)	0.001	1.056 (0.968-1.084)	0.464
**Postoperative FFP units[d]**	1.049 (1.007-1.097)	0.023	1.005 (0.952-1.060)	0.866

Results given as hazard ratios (HR) with 95% confidence intervals (95% CI). Factors showing significant results in the univariable analysis were included in the multivariable Cox regression model. Abbreviations used: *CAR*, C-reactive protein-to-albumin ratio; *CRP*, C-reactive protein; *Alb*, albumin; *BMI*, body mass index; *labMELD*, laboratory model of end-stage liver disease score; *BAR*, balance of risk; *SOFT*, survival outcomes following liver transplantation; *OLT*, orthotopic liver transplantation; *ICU*, intensive care unit; *RRT*, renal replacement therapy; *RBC*, red blood cell; *FFP*, fresh frozen plasma. [a] Refers to Schlegel et al. [b] Refers to Rana et al. [c] Refers to blood products given during the first 7 days following OLT.

Supplementary Table S4. Association of perioperative factors with 3-year recipient survival in patients with EAD and 90-day mortality

**Perioperative factor**	**Univariable Cox regression analysis**		**Multivariable Cox regression analysis**	
	HR (95% CI)	*p*-value	HR (95% CI)	*p*-value
**CAR>41%**	3.291 (2.141-5.058)	**<0.001**	2.025 (1.407-2.913)	**<0.001**
**Donor age (years)**	1.004 (0.992-1.017)	0.483		
**Donor BMI**	0.975 (0.944-1.007)	0.129		
**Donor sex (male)**	0.782 (0.526-1.162)	0.223		
**Cause of donor death**	1.148 (0.942-1.400)	0.171		
**Allocation type**	1.053 (0.616-1.713)	0.816		
**Recipient age**	1.023 (0.985-1.053)	0.365		
**Recipient BMI**	0.999 (0.985-1.013)	0.880		
**Recipient sex (male)**	0.582 (0.334-1.021)	0.072		
**Etiology of liver disease**	0.978 (0.899-1.063)	0.595		
**labMELD**	1.056 (1.037-1.075)	**<0.001**		
**BAR score[a]**	1.116 (1.078-1.155)	**<0.001**	1.013 (0.975-1.052)	0.517
**SOFT score[b]**	1.061 (1.043-1.080)	**<0.001**		
**Recipient pre-OLT ICU (yes)**	3.526 (2.359-5.271)	**<0.001**		
**Recipient pre-OLT abdominal surgery (yes)**	0.978 (0.635-1.504)	0.918		
**Recipient inpatient pre-OLT (yes)**	2.529 (1.696-3.771)	**<0.001**	1.037 (0.683-1.575)	0.864
**Recipient pre-OLT encephalopathy (yes)**	1.773 (1.191-2.640)	**0.005**		
**Recipient pre-OLT ascites (yes)**	1.339 (0.892-2.010)	0.159		
**Pre-OLT renal failure (yes)**	2.441 (1.642-3.629)	**<0.001**	1.412 (0.977-2.040)	0.066
**Pre-OLT RRT (yes)**	2.880 (1.865-4.446)	**<0.001**		
**Cold ischemia time (minutes)**	1.001 (1.000-1.003)	**0.058**		
**Warm ischemia time (minutes)**	1.026 (1.008-1.045)	**0.005**		
**Intraoperative RBC units**	1.046 (1.043-1.059)	**<0.001**		
**Intraoperative FFP units**	1.032 (1.019-1.046)	**<0.001**		
**Postoperative RBC units[c]**	1.059 (1.042-1.076)	**<0.001**	1.013 (0.983-1.043)	0.411
**Postoperative FFP units[c]**	1.042 (1.030-1.053)	**<0.001**	1.029 (1.008-1.050)	**0.006**

Results given as hazard ratios (HR) with 95% confidence intervals (95% CI). Factors showing significant results in the univariable analysis were included in the multivariable Cox regression model. To avoid multicollinearity, certain variables found to be significantly different in the subgroup analysis were not included in the multivariable Cox regression analysis (e.g., postoperative RBC and FFP, etc.). Abbreviations used: *CAR*, C-reactive-protein-to-albumin ratio; *CRP*, C-reactive protein; *Alb*, albumin; *BMI*, body mass index; *labMELD*, laboratory model of end-stage liver disease score; *BAR*, balance of risk; *SOFT*, survival outcomes following liver transplantation; *OLT*, orthotopic liver transplantation; *ICU*, intensive care unit; *RRT*, renal replacement therapy; *RBC*, red blood cell; *FFP*, fresh frozen plasma. [a] Refers to Schlegel et al. [b] Refers to Rana et al. [c] Refers to blood products given during the first 7 days following OLT.

Supplementary Table S5. Association of perioperative factors with 3-year graft survival in patients with EAD and 90-day mortality

Perioperative factor	Univariable Cox regression analysis		Multivariable Cox regression analysis	
	R (95% CI)	*p*-value	HR (95% CI)	*p*-value
**CAR>41%**	2.907 (1.960-4.311)	**<0.001**	2.242 (1.442-3.487)	**<0.001**
**Donor age (years)**	1.005 (0.993-1.016)	0.438		
**Donor BMI**	0.979 (0.950-1.008)	0.154		
**Donor sex (male)**	0.883 (0.507-1.533)	0.654		
**Cause of donor death**	1.028 (0.847-1.249)	0.777		
**Allocation type**	1.238 (0.884-1.732)	0.214		
**Recipient age**	1.013 (0.995-1.032)	0.162		
**Recipient BMI**	0.996 (0.978-1.015)	0.691		
**Recipient sex (male)**	0.579 (0.321-1.048)	0.079		
**Etiology of liver disease**	1.016 (0.942-1.096)	0.687		
**labMELD**	1.044 (1.027-1.062)	**<0.001**		
**BAR score[a]**	1.089 (1.054-1.124)	**<0.001**	1.018 (0.974-1.063)	0.430
**SOFT score[b]**	1.049 (1.032-1.067)	**<0.001**		
**Recipient pre-OLT ICU (yes)**	2.841 (1.936-4.168)	**<0.001**		
**Recipient pre-OLT abdominal surgery (yes)**	0.946 (0.632-1.416)	0.787		
**Recipient inpatient pre-OLT (yes)**	2.062 (1.423-2.989)	**<0.001**	1.145 (0.705-1.861)	0.585
**Recipient pre-OLT encephalopathy (yes)**	1.341 (0.921-1.953)	0.125		
**Recipient pre-OLT ascites (yes)**	1.357 (0.927-1.986)	0.116		
**Pre-OLT renal failure (yes)**	1.932 (1.328-2.809)	**<0.001**	1.120 (0.721-1.739)	0.615
**Pre-OLT RRT (yes)**	2.308 (1.512-3.525)	**<0.001**		
**Cold ischemia time (minutes)**	1.001 (0.999-1.002)	0.301		
**Warm ischemia time (minutes)**	1.013 (0.990-1.043)	0.219		
**Intraoperative RBC units**	1.040 (1.028-1.052)	**<0.001**		
**Intraoperative FFP units**	1.026 (1.014-1.040)	**<0.001**		
**Postoperative RBC units[c]**	1.059 (1.044-1.074)	**<0.001**	1.009 (0.976-1.042)	0.607
**Postoperative FFP units[c]**	1.044 (1.034-1.054)	**<0.001**	1.031 (1.008-1.055	**0.009**

Results given as hazard ratios (HR) with 95% confidence intervals (95% CI). Factors showing significant results in the univariable analysis were included in the multivariable Cox regression model. Abbreviations used: *CAR*, C-reactive protein-to-albumin ratio; *CRP*, C-reactive protein; *Alb*, albumin; *BMI*, body mass index; *labMELD*, laboratory model of end-stage liver disease score; *BAR*, balance of risk; *SOFT*, survival outcomes following liver transplantation; *OLT*, orthotopic liver transplantation; *ICU*, intensive care unit; *RRT*, renal replacement therapy; *RBC*, red blood cell; *FFP*, fresh frozen plasma. [a] Refers to Schlegel et al. [b] Refers to Rana et al. [c] Refers to blood products given during the first 7 days following OLT.

Supplementary Table S6. Association of perioperative factors with 5-year recipient survival in patients with EAD and 90-day mortality

**Perioperative factor**	**Univariable Cox regression analysis**		**Multivariable Cox regression analysis**	
	HR (95% CI)	*p*-value	HR (95% CI)	*p*-value
**CAR>41%**	2.695 (1.850-3.925)	<0.001	2.040 (1.415-2.942)	**<0.001**
**Donor age (years)**	1.007 (0.996-1.019)	0.218		
**Donor BMI**	0.984 (0.958-1.012)	0.258		
**Donor sex (male)**	0.848 (0.592-1.215)	0.370		
**Cause of donor death**	1.181 (0.987-1.413)	0.069		
**Allocation type**	1.358 (0.979-1.886)	0.067		
**Recipient age**	1.017 (0.999-1.036)	0.060		
**Recipient BMI**	0.998 (0.984-1.012)	0.796		
**Recipient sex (male)**	0.656 (0.454-0.947)	0.024		
**Etiology of liver disease**	0.964 (0.892-1.041)	0.349		
**labMELD**	1.045 (1.028-1.062)	<0.001		
**BAR score[a]**	1.093 (1.059-1.128)	<0.001	1.018 (0.980-1.058)	0.366
**SOFT score[b]**	1.050 (1.033-1.067)	<0.001		
**Recipient pre-OLT ICU (yes)**	2.767 (1.903-4.025)	<0.001		
**Recipient pre-OLT abdominal surgery (yes)**	0.935 (0.632-1.384)	0.737		
**Recipient inpatient pre-OLT (yes)**	1.864 (1.300-2.672)	<0.001	0.999 (0.658-1.518)	0.997
**Recipient pre-OLT encephalopathy (yes)**	1.475 (1.026-2.121)	0.036		
**Recipient pre-OLT ascites (yes)**	1.284 (0.889-1.854)	0.183		
**Pre-OLT renal failure (yes)**	1.800 (1.248-2.597)	0.002	1.305 (0.904-1.882)	0.155
**Pre-OLT RRT (yes)**	2.225 (1.464-3.383)	<0.001		
**Cold ischaemia time (minutes)**	1.000 (0.998-1.002)	0.805		
**Warm ischaemia time (minutes)**	1.020 (0.991-1.048)	0.241		
**Intraoperative RBC units**	1.044 (1.032-1.057)	<0.001		
**Intraoperative FFP units**	1.030 (1.017-1.043)	<0.001		
**Postoperative RBC units[d]**	1.057 (1.040-1.074)	<0.001	1.015 (0.985-1.045)	0.340
**Postoperative FFP units[d]**	1.040 (1.028-1.051)	0.024	1.027 (1.006-1.048)	**0.011**

Results given as hazard ratios (HR) with 95% confidence intervals (95% CI). Factors showing significant results in the univariable analysis were included in the multivariable Cox regression model. Abbreviations used: *CAR*, C-reactive protein-to-albumin ratio; *CRP*, C-reactive protein; *Alb*, albumin; *BMI*, body mass index; *labMELD*, laboratory model of end-stage liver disease score; *BAR*, balance of risk; *SOFT*, survival outcomes following liver transplantation; *OLT*, orthotopic liver transplantation; *ICU*, intensive care unit; *RRT*, renal replacement therapy; *RBC*, red blood cell; *FFP*, fresh frozen plasma. [a] Refers to Schlegel et al. [b] Refers to Rana et al. [c] Refers to blood products given during the first 7 days following OLT.

Supplementary Table S7. Association of perioperative factors with 5-year graft survival in patients with EAD and 90-day mortality

**Perioperative factor**	**Univariable Cox regression analysis**		**Multivariable Cox regression analysis**	
	HR (95% CI)	*p*-value	HR (95% CI)	*p*-value
**CAR>41%**	2.507 (1.762-3.566)	<0.001	2.180 (1.470-3.234)	**<0.001**
**Donor age (years)**	1.008 (0.997-1.019)	0.168		
**Donor BMI**	0.988 (0.963-1.013)	0.344		
**Donor sex (male)**	0.763 (0.543-1.072)	0.119		
**Cause of donor death**	1.070 (0.897-1.277)	0.454		
**Allocation type**	1.201 (0.886-1.628)	0.238		
**Recipient age**	1.007 (0.991-1.024)	0.367		
**Recipient BMI**	0.995 (0.977-1.014)	0.618		
**Recipient sex (male)**	0.696 (0.491-1.078)	0.186		
**Etiology of liver disease**	0.827 (0.924-1.065)	0.827		
**labMELD**	1.035 (1.019-1.051)	<0.001		
**BAR score**[a]	1.069 (1.038-1.101)	<0.001	1.017 (0.976-1.060)	0.414
**SOFT score**[b]	1.023 (1.023-1.057)	<0.001		
**Recipient pre-OLT ICU (yes)**	2.344 (1.632-3.366)	<0.001		
**Recipient pre-OLT abdominal surgery (yes)**	0.907 (0.626-1.315)	0.606		
**Recipient inpatient pre-OLT (yes)**	1.619 (1.150-2.888)	0.006	0.968 (0.618-1.515)	0.885
**Recipient pre-OLT encephalopathy (yes)**	1.173 (0.828-1.661)	0.369		
**Recipient pre-OLT ascites (yes)**	1.269 (0.897-1.796)	0.179		
**Pre-OLT renal failure (yes)**	1.502 (1.056-2.137)	0.024	0.930 (0.616-1.406)	0.350
**Pre-OLT RRT (yes)**	1.848 (1.224-2.789)	0.003		
**Cold ischaemia time (minutes)**	1.001 (0.999-1.002)	0.230		
**Warm ischaemia time (minutes)**	1.015 (0.998-1.033)	0.080		
**Intraoperative RBC units**	1.039 (1.028-1.051)	<0.001		
**Intraoperative FFP units**	1.025 (1.015-1.038)	<0.001		
**Postoperative RBC units**[d]	1.058 (1.043-1.073)	<0.001	1.015 (0.984-1.048)	0.350
**Postoperative FFP units**[d]	1.043 (1.032-1.053)	<0.001	1.028 (1.005-1.051)	**0.015**

Results given as hazard ratios (HR) with 95% confidence intervals (95% CI). Factors showing significant results in the univariable analysis were included in the multivariable Cox regression model. Abbreviations used: *CAR*, C-reactive protein-to-albumin ratio; *CRP*, C-reactive protein; *Alb*, albumin; *BMI*, body mass index; *labMELD*, laboratory model of end-stage liver disease score; *BAR*, balance of risk; *SOFT*, survival outcomes following liver transplantation; *OLT*, orthotopic liver transplantation; *ICU*, intensive care unit; *RRT*, renal replacement therapy; *RBC*, red blood cell; *FFP*, fresh frozen plasma. [a] Refers to Schlegel et al. [b] Refers to Rana et al. [c] Refers to blood products given during the first 7 days following OLT.

Supplementary Table S8. Association of perioperative factors with 3-year recipient survival (incl. MELD score multivariable analysis)

Perioperative factor	Univariable Cox regression analysis		Multivariable Cox regression analysis	
	HR (95% CI)	*p*-value	HR (95% CI)	*p*-value
**CAR>41%**	3.902 (2.015-7.555)	**<0.001**	**2.082 (1.262-3.435)**	**0.004**
**Donor age (years)**	0.998 (0.980-1.016)	0.828		
**Donor BMI**	0.968 (0.918-1.020)	0.193		
**Donor sex (male)**	1.027 (0.572-1.844)	0.930		
**Cause of donor death**	1.020 (0.749-1.391)	0.899		
**Allocation type**	1.209 (0.721-2.026)	0.468		
**Recipient age**	1.020 (0.990-1.050)	0.181		
**Recipient BMI**	0.998 (0.977-1.019)	0.840		
**Recipient sex (male)**	0.533 (0.296-0.960)	**0.040**		
**Etiology of liver disease**	1.004 (0.887-1.136)	0.950		
**labMELD**	1.047 (1.020-1.075)	**<0.001**	1.000 (0.971-1.030)	0.995
**BAR score[a]**	1.087 (1.028-1.148)	**0.003**		
**SOFT score[b]**	1.046 (1.014-1.080)	**0.004**		
**Recipient pre-OLT ICU (yes)**	3.018 (1.639-5.559)	**<0.001**		
**Recipient pre-OLT abdominal surgery (yes)**	0.662 (0.328-1.338)	0.234		
**Recipient inpatient pre-OLT (yes)**	2.445 (1.357-4.403)	**0.003**	1.089 (0.599-1.979)	0.780
**Recipient pre-OLT encephalopathy (yes)**	1.855 (1.034-3.328)	**0.039**		
**Recipient pre-OLT ascites (yes)**	1.207 (0.665-2.191)	0.535		
**Pre-OLT renal failure (yes)**	2.291 (1.272-4.126)	**0.006**	1.348 (0.815-2.231)	0.245
**Pre-OLT RRT (yes)**	2.618 (1.326-5.169)	**0.011**		
**Cold ischemia time (minutes)**	1.000 (0.997-1.002)	0.967		
**Warm ischemia time (minutes)**	1.024 (0.994-1.056)	0.129		
**Intraoperative RBC units**	1.047 (1.022-1.073)	**0.002**		
**Intraoperative FFP units**	1.014 (0.987-1.041)	0.314		
**Postoperative RBC units[c]**	1.088 (1.042-1.137)	**<0.001**	1.034 (0.815-2.231)	0.249
**Postoperative FFP units[c]**	1.067 (1.019-1.117)	**0.005**	1.030 (0.984-1.077)	0.204

Results given as hazard ratios (HR) with 95% confidence intervals (95% CI). Factors showing significant results in the univariable analysis were included in the multivariable Cox regression model. To avoid multicollinearity, certain variables found to be significantly different in the subgroup analysis were not included in the multivariable Cox regression analysis (e.g., postoperative RBC and FFP, etc.). Abbreviations used: *CAR*, C-reactive protein-to-albumin ratio; *CRP*, C-reactive protein; *Alb*, albumin; *BMI*, body mass index; *labMELD*, laboratory model of end-stage liver disease score; *BAR*, balance of risk; *SOFT*, survival outcomes following liver transplantation; *OLT*, orthotopic liver transplantation; *ICU*, intensive care unit; *RRT*, renal replacement therapy; *RBC*, red blood cell; *FFP*, fresh frozen plasma. [a] Refers to Schlegel et al. [b] Refers to Rana et al. [c] Refers to blood products given during the first 7 days following OLT.

Supplementary Table S9. Association of perioperative factors with 3-year graft survival (incl. MELD score multivariable analysis)

Perioperative factor	Univariable Cox regression analysis		Multivariable Cox regression analysis	
	R (95% CI)	*p*-value	HR (95% CI)	*p*-value
**CAR>41%**	4.605 (2.406-8.813)	**<0.001**	**3.573 (1.754–7.277)**	**<0.001**
**Donor age (years)**	1.001 (0.984-1.018)	0.918		
**Donor BMI**	0.987 (0.944-1.031)	0.536		
**Donor sex (male)**	0.880 (0.505-1.531)	0.650		
**Cause of donor death**	0.928 (0.682-1.262)	0.633		
**Allocation type**	1.048 (0.647-1.697)	0.848		
**Recipient age**	1.014 (0.987-1.042)	0.305		
**Recipient BMI**	0.995 (0.968-1.023)	0.671		
**Recipient sex (male)**	0.589 (0.336-1.033)	0.070		
**Etiology of liver disease**	1.008 (0.898-1.133)	0.888		
**labMELD**	1.045 (1.019-1.071)	**<0.001**	1.009 (0.973–1.046)	0.631
**BAR score**[a]	1.074 (1.019-1.132)	**0.009**		
**SOFT score**[b]	1.043 (1.012-1.074)	**0.010**		
**Recipient pre-OLT ICU (yes)**	2.872 (1.599-5.158)	**<0.001**		
**Recipient pre-OLT abdominal surgery (yes)**	0.646 (0.331-1.262)	0.183		
**Recipient inpatient pre-OLT (yes)**	2.545 (1.455-4.451)	**0.001**	1.400 (0.670–2.923)	0.371
**Recipient pre-OLT encephalopathy (yes)**	1.496 (0.858-2.610)	0.159		
**Recipient pre-OLT ascites (yes)**	1.461 (0.820-2.603)	0.191		
**Pre-OLT renal failure (yes)**	1.869 (1.061-3.292)	**0.035**	0.959 (0.506–1.817)	0.897
**Pre-OLT RRT (yes)**	2.238 (1.146-4.372)	**0.029**		
**Cold ischemia time (minutes)**	0.999 (0.997-1.002)	0.633		
**Warm ischemia time (minutes)**	1.022 (0.993-1.052)	0.148		
**Intraoperative RBC units**	1.043 (1.018-1.070)	**0.005**		
**Intraoperative FFP units**	1.004 (0.976-1.033)	0.776		
**Postoperative RBC units**[c]	1.082 (1.036-1.129)	**0.003**	**1.071 (1.002–1.144)**	**0.044**
**Postoperative FFP units**[c]	1.067 (1.022-1.114)	**0.008**	1.019 (0.964–1.077)	0.509

Results given as hazard ratios (HR) with 95% confidence intervals (95% CI). Factors showing significant results in the univariable analysis were included in the multivariable Cox regression model. Abbreviations used: *CAR*, C-reactive protein-to-albumin ratio; *CRP*, C-reactive protein; *Alb*, albumin; *BMI*, body mass index; *labMELD*, laboratory model of end-stage liver disease score; *BAR*, balance of risk; *SOFT*, survival outcomes following liver transplantation; *OLT*, orthotopic liver transplantation; *ICU*, intensive care unit; *RRT*, renal replacement therapy; *RBC*, red blood cell; *FFP*, fresh frozen plasma. [a] Refers to Schlegel et al. [b] Refers to Rana et al. [c] Refers to blood products given during the first 7 days following OLT.

Supplementary Table S10. Association of perioperative factors with 5-year recipient survival (incl. MELD score multivariable analysis)

Perioperative factor	Univariable Cox regression analysis		Multivariable Cox regression analysis	
	HR (95% CI)	*p*-value	HR (95% CI)	*p*-value
**CAR>41%**	2.775 (1.596-4.826)	<0.001	**2.076 (1.254–3.435)**	**0.004**
**Donor age (years)**	1.005 (0.989-1.022)	0.523		
**Donor BMI**	0.969 (0.926-1.015)	0.185		
**Donor sex (male)**	1.064 (0.629-1.799)	0.817		
**Cause of donor death**	1.140 (0.873-1.489)	0.334		
**Allocation type**	1.125 (0.715-1.772)	0.610		
**Recipient age**	1.012 (0.987-1.038)	0.360		
**Recipient BMI**	0.993 (0.963-1.025)	0.678		
**Recipient sex (male)**	0.617 (0.362-1.050)	0.075		
**Etiology of liver disease**	0.985 (0.880-1.102)	0.787		
**labMELD**	1.038 (1.013-1.063)	0.002	1.004 (0.974–1.035)	0.793
**BAR score[a]**	1.064 (1.013-1.118)	0.014		
**SOFT score[b]**	1.035 (1.004-1.066)	0.024		
**Recipient pre-OLT ICU (yes)**	2.361 (1.335-4.174)	0.003		
**Recipient pre-OLT abdominal surgery (yes)**	0.545 (0.282-1.054)	0.071		
**Recipient inpatient pre-OLT (yes)**	1.738 (1.028-2.940)	0.039	1.026 (0.565–1.863)	0.932
**Recipient pre-OLT encephalopathy (yes)**	1.426 (0.842-2.416)	0.187		
**Recipient pre-OLT ascites (yes)**	1.140 (0.669-1.941)	0.630		
**Pre-OLT renal failure (yes)**	1.720 (1.001-2.955)	0.050	1.269 (0.772–2.088)	0.348
**Pre-OLT RRT (yes)**	2.003 (1.036-3.874)	0.039		
**Cold ischaemia time (minutes)**	1.000 (0.998-1.002)	0.815		
**Warm ischaemia time (minutes)**	1.020 (0.991-1.050)	0.186		
**Intraoperative RBC units**	1.042 (1.016-1.068)	0.001		
**Intraoperative FFP units**	1.012 (0.986-1.037)	0.371		
**Postoperative RBC units[d]**	1.079 (1.035-1.124)	<0.001	1.269 (0.772–2.088)	0.348
**Postoperative FFP units[d]**	1.051 (1.007-1.098)	0.024	1.034 (0.978–1.094)	0.236

Results given as hazard ratios (HR) with 95% confidence intervals (95% CI). Factors showing significant results in the univariable analysis were included in the multivariable Cox regression model. Abbreviations used: *CAR*, C-reactive protein-to-albumin ratio; *CRP*, C-reactive protein; *Alb*, albumin; *BMI*, body mass index; *labMELD*, laboratory model of end-stage liver disease score; *BAR*, balance of risk; *SOFT*, survival outcomes following liver transplantation; *OLT*, orthotopic liver transplantation; *ICU*, intensive care unit; *RRT*, renal replacement therapy; *RBC*, red blood cell; *FFP*, fresh frozen plasma. [a] Refers to Schlegel et al. [b] Refers to Rana et al. [c] Refers to blood products given during the first 7 days following OLT.

Supplementary Table S11. Association of perioperative factors with 5-year graft survival (incl. MELD score multivariable analysis)

Perioperative factor	Univariable Cox analysis		Multivariable Cox regression analysis	
	HR (95% CI)	*p*-value	HR (95% CI)	*p*-value
**CAR>41%**	3.183 (1.872-5.412)	<0.001	**2.762 (1.535–4.972)**	**0.001**
**Donor age (years)**	1.009 (0.994-1.026)	0.246		
**Donor BMI**	0.986 (0.949-1.025)	0.490		
**Donor sex (male)**	0.930 (0.568-1.525)	0.774		
**Cause of donor death**	1.022 (0.785-1.330)	0.874		
**Allocation type**	1.020 (0.668-1.557)	0.926		
**Recipient age**	1.006 (0.983-1.030)	0.601		
**Recipient BMI**	0.987 (0.941-1.034)	0.575		
**Recipient sex (male)**	0.657 (0.397-1.089)	0.103		
**Etiology of liver disease**	0.988 (0.889-1.097)	0.815		
**labMELD**	1.034 (1.011-1.058)	0.004	1.010 (0.977–1.044)	0.566
**BAR score[a]**	1.049 (1.001-1.100)	0.045		
**SOFT score[b]**	1.031 (1.003-1.060)	0.032		
**Recipient pre-OLT ICU (yes)**	2.214 (1.281-3.825)	0.004		
**Recipient pre-OLT abdominal surgery (yes)**	0.569 (0.309-1.048)	0.071		
**Recipient inpatient pre-OLT (yes)**	1.759 (1.071-2.888)	0.026	1.023 (0.526–1.990)	0.946
**Recipient pre-OLT encephalopathy (yes)**	1.146 (0.691-1.898)	0.598		
**Recipient pre-OLT ascites (yes)**	1.329 (0.799-2.210)	0.273		
**Pre-OLT renal failure (yes)**	1.395 (0.826-2.356)	0.213		
**Pre-OLT RRT (yes)**	1.693 (0.883-3.246)	0.113		
**Cold ischaemia time (minutes)**	1.000 (0.998-1.002)	0.763		
**Warm ischaemia time (minutes)**	1.016 (0.988-1.044)	0.266		
**Intraoperative RBC units**	1.037 (1.011-1.064)	0.005		
**Intraoperative FFP units**	1.002 (0.975-1.029)	0.902		
**Postoperative RBC units[d]**	1.070 (1.027-1.115)	0.001	**1.064 (1.000–1.131)**	**0.048**
**Postoperative FFP units[d]**	1.049 (1.007-1.097)	0.023	1.005 (0.953–1.061)	0.844

Results given as hazard ratios (HR) with 95% confidence intervals (95% CI). Factors showing significant results in the univariable analysis were included in the multivariable Cox regression model. Abbreviations used: *CAR*, C-reactive protein-to-albumin ratio; *CRP*, C-reactive protein; *Alb*, albumin; *BMI*, body mass index; *labMELD*, laboratory model of end-stage liver disease score; *BAR*, balance of risk; *SOFT*, survival outcomes following liver transplantation; *OLT*, orthotopic liver transplantation; *ICU*, intensive care unit; *RRT*, renal replacement therapy; *RBC*, red blood cell; *FFP*, fresh frozen plasma. [a] Refers to Schlegel et al. [b] Refers to Rana et al. [c] Refers to blood products given during the first 7 days following OLT.

Supplementary Table S12. Association of perioperative factors with 3-year recipient survival (incl. SOFT score multivariable analysis)

**Perioperative factor**	**Univariable Cox regression analysis**		**Multivariable Cox regression analysis**	
	HR (95% CI)	*p*-value	HR (95% CI)	*p*-value
**CAR>41%**	3.902 (2.015-7.555)	**<0.001**	**1.987 (1.198–3.296)**	**0.008**
**Donor age (years)**	0.998 (0.980-1.016)	0.828		
**Donor BMI**	0.968 (0.918-1.020)	0.193		
**Donor sex (male)**	1.027 (0.572-1.844)	0.930		
**Cause of donor death**	1.020 (0.749-1.391)	0.899		
**Allocation type**	1.209 (0.721-2.026)	0.468		
**Recipient age**	1.020 (0.990-1.050)	0.181		
**Recipient BMI**	0.998 (0.977-1.019)	0.840		
**Recipient sex (male)**	0.533 (0.296-0.960)	**0.040**		
**Etiology of liver disease**	1.004 (0.887-1.136)	0.950		
**labMELD**	1.047 (1.020-1.075)	**<0.001**		
**BAR score[a]**	1.087 (1.028-1.148)	**0.003**		
**SOFT score[b]**	1.046 (1.014-1.080)	**0.004**	1.004 (0.968–1.041)	0.847
**Recipient pre-OLT ICU (yes)**	3.018 (1.639-5.559)	**<0.001**		
**Recipient pre-OLT abdominal surgery (yes)**	0.662 (0.328-1.338)	0.234		
**Recipient inpatient pre-OLT (yes)**	2.445 (1.357-4.403)	**0.003**	0.930 (0.496–1.742)	0.820
**Recipient pre-OLT encephalopathy (yes)**	1.855 (1.034-3.328)	**0.039**		
**Recipient pre-OLT ascites (yes)**	1.207 (0.665-2.191)	0.535		
**Pre-OLT renal failure (yes)**	2.291 (1.272-4.126)	**0.006**	1.471 (0.870–2.485)	0.150
**Pre-OLT RRT (yes)**	2.618 (1.326-5.169)	**0.011**		
**Cold ischemia time (minutes)**	1.000 (0.997-1.002)	0.967		
**Warm ischemia time (minutes)**	1.024 (0.994-1.056)	0.129		
**Intraoperative RBC units**	1.047 (1.022-1.073)	**0.002**		
**Intraoperative FFP units**	1.014 (0.987-1.041)	0.314		
**Postoperative RBC units[c]**	1.088 (1.042-1.137)	**<0.001**	1.026 (0.966–1.090)	0.405
**Postoperative FFP units[c]**	1.067 (1.019-1.117)	**0.005**	1.022 (0.974–1.073)	0.368

Results given as hazard ratios (HR) with 95% confidence intervals (95% CI). Factors showing significant results in the univariable analysis were included in the multivariable Cox regression model. To avoid multicollinearity, certain variables found to be significantly different in the subgroup analysis were not included in the multivariable Cox regression analysis (e.g., postoperative RBC and FFP, etc.). Abbreviations used: *CAR*, C-reactive-protein-to-albumin ratio; *CRP*, C-reactive protein; *Alb*, Albumin; *BMI*, body mass index; *labMELD*, laboratory model of end-stage liver disease score; *BAR*, balance of risk; *SOFT*, survival outcomes following liver transplantation; *OLT*, orthotopic liver transplantation; *ICU*, intensive care unit; *RRT*, renal replacement therapy; *RBC*, red blood cell; *FFP*, fresh frozen plasma. [a] Refers to Schlegel et al. [b] Refers to Rana et al. [c] Refers to blood products given during the first 7 days following OLT.

Supplementary Table S13. Association of perioperative factors with 3-year graft survival (incl. SOFT score multivariable analysis)

Perioperative factor	Univariable Cox regression analysis		Multivariable Cox regression analysis	
	R (95% CI)	*p*-value	HR (95% CI)	*p*-value
**CAR>41%**	4.605 (2.406-8.813)	**<0.001**	**3.505 (1.677–7.328)**	**0.001**
**Donor age (years)**	1.001 (0.984-1.018)	0.918		
**Donor BMI**	0.987 (0.944-1.031)	0.536		
**Donor sex (male)**	0.880 (0.505-1.531)	0.650		
**Cause of donor death**	0.928 (0.682-1.262)	0.633		
**Allocation type**	1.048 (0.647-1.697)	0.848		
**Recipient age**	1.014 (0.987-1.042)	0.305		
**Recipient BMI**	0.995 (0.968-1.023)	0.671		
**Recipient sex (male)**	0.589 (0.336-1.033)	0.070		
**Etiology of liver disease**	1.008 (0.898-1.133)	0.888		
**labMELD**	1.045 (1.019-1.071)	**<0.001**		
**BAR score[a]**	1.074 (1.019-1.132)	**0.009**		
**SOFT score[b]**	1.043 (1.012-1.074)	**0.010**	1.000 (0.958–1.045)	0.983
**Recipient pre-OLT ICU (yes)**	2.872 (1.599-5.158)	**<0.001**		
**Recipient pre-OLT abdominal surgery (yes)**	0.646 (0.331-1.262)	0.183		
**Recipient inpatient pre-OLT (yes)**	2.545 (1.455-4.451)	**0.001**	1.298 (0.593–2.839)	0.514
**Recipient pre-OLT encephalopathy (yes)**	1.496 (0.858-2.610)	0.159		
**Recipient pre-OLT ascites (yes)**	1.461 (0.820-2.603)	0.191		
**Pre-OLT renal failure (yes)**	1.869 (1.061-3.292)	**0.035**	1.139 (0.575–2.259)	0.709
**Pre-OLT RRT (yes)**	2.238 (1.146-4.372)	**0.029**		
**Cold ischemia time (minutes)**	0.999 (0.997-1.002)	0.633		
**Warm ischemia time (minutes)**	1.022 (0.993-1.052)	0.148		
**Intraoperative RBC units**	1.043 (1.018-1.070)	**0.005**		
**Intraoperative FFP units**	1.004 (0.976-1.033)	0.776		
**Postoperative RBC units[c]**	1.082 (1.036-1.129)	**0.003**	1.064 (0.988–1.147)	0.102
**Postoperative FFP units[c]**	1.067 (1.022-1.114)	**0.008**	1.002 (0.941–1.067)	0.944

Results given as hazard ratios (HR) with 95% confidence intervals (95% CI). Factors showing significant results in the univariable analysis were included in the multivariable Cox regression model. Abbreviations used: *CAR*, C-reactive protein-to-albumin ratio; *CRP*, C-reactive protein; *Alb*, albumin; *BMI*, body mass index; *labMELD*, laboratory model of end-stage liver disease score; *BAR*, balance of risk; *SOFT*, survival outcomes following liver transplantation; *OLT*, orthotopic liver transplantation; *ICU*, intensive care unit; *RRT*, renal replacement therapy; *RBC*, red blood cell; *FFP*, fresh frozen plasma. [a] Refers to Schlegel et al. [b] Refers to Rana et al. [c] Refers to blood products given during the first 7 days following OLT.

Supplementary Table S14. Association of perioperative factors with 5-year recipient survival (incl. SOFT score multivariable analysis)

Perioperative factor	Univariable Cox regression analysis		Multivariable Cox regression analysis	
	HR (95% CI)	*p*-value	HR (95% CI)	*p*-value
**CAR>41%**	2.775 (1.596-4.826)	<0.001	**1.997 (1.199–3.325)**	**0.008**
**Donor age (years)**	1.005 (0.989-1.022)	0.523		
**Donor BMI**	0.969 (0.926-1.015)	0.185		
**Donor sex (male)**	1.064 (0.629-1.799)	0.817		
**Cause of donor death**	1.140 (0.873-1.489)	0.334		
**Allocation type**	1.125 (0.715-1.772)	0.610		
**Recipient age**	1.012 (0.987-1.038)	0.360		
**Recipient BMI**	0.993 (0.963-1.025)	0.678		
**Recipient sex (male)**	0.617 (0.362-1.050)	0.075		
**Etiology of liver disease**	0.985 (0.880-1.102)	0.787		
**labMELD**	1.038 (1.013-1.063)	0.002		
**BAR score**[a]	1.064 (1.013-1.118)	0.014		
**SOFT score**[b]	1.035 (1.004-1.066)	0.024	1.009 (0.973–1.047)	0.635
**Recipient pre-OLT ICU (yes)**	2.361 (1.335-4.174)	0.003		
**Recipient pre-OLT abdominal surgery (yes)**	0.545 (0.282-1.054)	0.071		
**Recipient inpatient pre-OLT (yes)**	1.738 (1.028-2.940)	0.039	0.871 (0.462–1.640)	0.668
**Recipient pre-OLT encephalopathy (yes)**	1.426 (0.842-2.416)	0.187		
**Recipient pre-OLT ascites (yes)**	1.140 (0.669-1.941)	0.630		
**Pre-OLT renal failure (yes)**	1.720 (1.001-2.955)	0.050	1.372 (0.819–2.298)	0.230
**Pre-OLT RRT (yes)**	2.003 (1.036-3.874)	0.039		
**Cold ischaemia time (minutes)**	1.000 (0.998-1.002)	0.815		
**Warm ischaemia time (minutes)**	1.020 (0.991-1.050)	0.186		
**Intraoperative RBC units**	1.042 (1.016-1.068)	0.001		
**Intraoperative FFP units**	1.012 (0.986-1.037)	0.371		
**Postoperative RBC units**[d]	1.079 (1.035-1.124)	<0.001	1.026 (0.966–1.090)	0.398
**Postoperative FFP units**[d]	1.051 (1.007-1.098)	0.024	1.018 (0.971–1.068)	0.463

Results given as hazard ratios (HR) with 95% confidence intervals (95% CI). Factors showing significant results in the univariable analysis were included in the multivariable Cox regression model. Abbreviations used: *CAR*, C-reactive protein-to-albumin ratio; *CRP*, C-reactive protein; *Alb*, albumin; *BMI*, body mass index; *labMELD*, laboratory model of end-stage liver disease score; *BAR*, balance of risk; *SOFT*, survival outcomes following liver transplantation; *OLT*, orthotopic liver transplantation; *ICU*, intensive care unit; *RRT*, renal replacement therapy; *RBC*, red blood cell; *FFP*, fresh frozen plasma. [a] Refers to Schlegel et al. [b] Refers to Rana et al. [c] Refers to blood products given during the first 7 days following OLT.

Supplementary Table S15. Association of perioperative factors with 5-year graft survival (incl. SOFT score multivariable analysis)

**Perioperative factor**	**Univariable Cox analysis**		**Multivariable Cox regression analysis**	
	HR (95% CI)	*p*-value	HR (95% CI)	*p*-value
**CAR>41%**	3.183 (1.872-5.412)	<0.001	**2.719 (1.494–4.950)**	**0.001**
**Donor age (years)**	1.009 (0.994-1.026)	0.246		
**Donor BMI**	0.986 (0.949-1.025)	0.490		
**Donor sex (male)**	0.930 (0.568-1.525)	0.774		
**Cause of donor death**	1.022 (0.785-1.330)	0.874		
**Allocation type**	1.020 (0.668-1.557)	0.926		
**Recipient age**	1.006 (0.983-1.030)	0.601		
**Recipient BMI**	0.987 (0.941-1.034)	0.575		
**Recipient sex (male)**	0.657 (0.397-1.089)	0.103		
**Etiology of liver disease**	0.988 (0.889-1.097)	0.815		
**labMELD**	1.034 (1.011-1.058)	0.004		
**BAR score**[a]	1.049 (1.001-1.100)	0.045		
**SOFT score**[b]	1.031 (1.003-1.060)	0.032	1.006 (0.966–1.047)	0.778
**Recipient pre-OLT ICU (yes)**	2.214 (1.281-3.825)	0.004		
**Recipient pre-OLT abdominal surgery (yes)**	0.569 (0.309-1.048)	0.071		
**Recipient inpatient pre-OLT (yes)**	1.759 (1.071-2.888)	0.026	0.960 (0.473–1.949)	0.910
**Recipient pre-OLT encephalopathy (yes)**	1.146 (0.691-1.898)	0.598		
**Recipient pre-OLT ascites (yes)**	1.329 (0.799-2.210)	0.273		
**Pre-OLT renal failure (yes)**	1.395 (0.826-2.356)	0.213		
**Pre-OLT RRT (yes)**	1.693 (0.883-3.246)	0.113		
**Cold ischaemia time (minutes)**	1.000 (0.998-1.002)	0.763		
**Warm ischaemia time (minutes)**	1.016 (0.988-1.044)	0.266		
**Intraoperative RBC units**	1.037 (1.011-1.064)	0.005		
**Intraoperative FFP units**	1.002 (0.975-1.029)	0.902		
**Postoperative RBC units**[d]	1.070 (1.027-1.115)	0.001	1.061 (0.991–1.136)	0.087
**Postoperative FFP units**[d]	1.049 (1.007-1.097)	0.023	0.992 (0.934–1.053)	0.782

Results given as hazard ratios (HR) with 95% confidence intervals (95% CI). Factors showing significant results in the univariable analysis were included in the multivariable Cox regression model. Abbreviations used: *CAR*, C-reactive protein-to-albumin ratio; *CRP*, C-reactive protein; *Alb*, albumin; *BMI*, body mass index; *labMELD*, laboratory model of end-stage liver disease score; *BAR*, balance of risk; *SOFT*, survival outcomes following liver transplantation; *OLT*, orthotopic liver transplantation; *ICU*, intensive care unit; *RRT*, renal replacement therapy; *RBC*, red blood cell; *FFP*, fresh frozen plasma. [a] Refers to Schlegel et al. [b] Refers to Rana et al. [c] Refers to blood products given during the first 7 days following OLT.

Supplementary Table S16. Association of perioperative factors with 3-year recipient survival in patients with EAD and 90-day mortality (incl. MELD score multivariable analysis)

**Perioperative factor**	**Univariable Cox regression analysis**		**Multivariable Cox regression analysis**	
	HR (95% CI)	*p*-value	HR (95% CI)	*p*-value
**CAR>41%**	3.291 (2.141-5.058)	**<0.001**	**1.941 (1.347–2.798)**	**<0.001**
**Donor age (years)**	1.004 (0.992-1.017)	0.483		
**Donor BMI**	0.975 (0.944-1.007)	0.129		
**Donor sex (male)**	0.782 (0.526-1.162)	0.223		
**Cause of donor death**	1.148 (0.942-1.400)	0.171		
**Allocation type**	1.053 (0.616-1.713)	0.816		
**Recipient age**	1.023 (0.985-1.053)	0.365		
**Recipient BMI**	0.999 (0.985-1.013)	0.880		
**Recipient sex (male)**	0.582 (0.334-1.021)	0.072		
**Etiology of liver disease**	0.978 (0.899-1.063)	0.595		
**labMELD**	1.056 (1.037-1.075)	**<0.001**	1.018 (0.995–1.040)	0.122
**BAR score[a]**	1.116 (1.078-1.155)	**<0.001**		
**SOFT score[b]**	1.061 (1.043-1.080)	**<0.001**		
**Recipient pre-OLT ICU (yes)**	3.526 (2.359-5.271)	**<0.001**		
**Recipient pre-OLT abdominal surgery (yes)**	0.978 (0.635-1.504)	0.918		
**Recipient inpatient pre-OLT (yes)**	2.529 (1.696-3.771)	**<0.001**	0.897 (0.569–1.416)	0.641
**Recipient pre-OLT encephalopathy (yes)**	1.773 (1.191-2.640)	**0.005**		
**Recipient pre-OLT ascites (yes)**	1.339 (0.892-2.010)	0.159		
**Pre-OLT renal failure (yes)**	2.441 (1.642-3.629)	**<0.001**	1.364 (0.950–1.958)	0.092
**Pre-OLT RRT (yes)**	2.880 (1.865-4.446)	**<0.001**		
**Cold ischemia time (minutes)**	1.001 (1.000-1.003)	**0.058**		
**Warm ischemia time (minutes)**	1.026 (1.008-1.045)	**0.005**		
**Intraoperative RBC units**	1.046 (1.043-1.059)	**<0.001**		
**Intraoperative FFP units**	1.032 (1.019-1.046)	**<0.001**		
**Postoperative RBC units[c]**	1.059 (1.042-1.076)	**<0.001**	1.013 (0.983–1.043)	0.408
**Postoperative FFP units[c]**	1.042 (1.030-1.053)	**<0.001**	1.029 (1.008-1.050)	**0.006**

Results given as hazard ratios (HR) with 95% confidence intervals (95% CI). Factors showing significant results in the univariable analysis were included in the multivariable Cox regression model. To avoid multicollinearity, certain variables found to be significantly different in the subgroup analysis were not included in the multivariable Cox regression analysis (e.g., postoperative RBC and FFP, etc.). Abbreviations used: *CAR*, C-reactive protein-to-albumin ratio; *CRP*, C-reactive protein; *Alb*, albumin; *BMI*, body mass index; *labMELD*, laboratory model of end-stage liver disease score; *BAR*, balance of risk; *SOFT*, survival outcomes following liver transplantation; *OLT*, orthotopic liver transplantation; *ICU*, intensive care unit; *RRT*, renal replacement therapy; *RBC*, red blood cell; *FFP*, fresh frozen plasma. [a] Refers to Schlegel et al. [b] Refers to Rana et al. [c] Refers to blood products given during the first 7 days following OLT.

Supplementary Table S17. Association of perioperative factors with 3-year graft survival in patients with EAD and 90-day mortality (incl. MELD score multivariable analysis)

**Perioperative factor**	**Univariable Cox regression analysis**		**Multivariable Cox regression analysis**	
	R (95% CI)	*p*-value	HR (95% CI)	*p*-value
**CAR>41%**	2.907 (1.960-4.311)	**<0.001**	**2.112 (1.354–3.294)**	**0.001**
**Donor age (years)**	1.005 (0.993-1.016)	0.438		
**Donor BMI**	0.979 (0.950-1.008)	0.154		
**Donor sex (male)**	0.883 (0.507-1.533)	0.654		
**Cause of donor death**	1.028 (0.847-1.249)	0.777		
**Allocation type**	1.238 (0.884-1.732)	0.214		
**Recipient age**	1.013 (0.995-1.032)	0.162		
**Recipient BMI**	0.996 (0.978-1.015)	0.691		
**Recipient sex (male)**	0.579 (0.321-1.048)	0.079		
**Etiology of liver disease**	1.016 (0.942-1.096)	0.687		
**labMELD**	1.044 (1.027-1.062)	**<0.001**	1.022 (0.997–1.049)	0.091
**BAR score[a]**	1.089 (1.054-1.124)	**<0.001**		
**SOFT score[b]**	1.049 (1.032-1.067)	**<0.001**		
**Recipient pre-OLT ICU (yes)**	2.841 (1.936-4.168)	**<0.001**		
**Recipient pre-OLT abdominal surgery (yes)**	0.946 (0.632-1.416)	0.787		
**Recipient inpatient pre-OLT (yes)**	2.062 (1.423-2.989)	**<0.001**	0.966 (0.568–1.643)	0.900
**Recipient pre-OLT encephalopathy (yes)**	1.341 (0.921-1.953)	0.125		
**Recipient pre-OLT ascites (yes)**	1.357 (0.927-1.986)	0.116		
**Pre-OLT renal failure (yes)**	1.932 (1.328-2.809)	**<0.001**	1.074 (0.695–1.659)	0.747
**Pre-OLT RRT (yes)**	2.308 (1.512-3.525)	**<0.001**		
**Cold ischemia time (minutes)**	1.001 (0.999-1.002)	0.301		
**Warm ischemia time (minutes)**	1.013 (0.990-1.043)	0.219		
**Intraoperative RBC units**	1.040 (1.028-1.052)	**<0.001**		
**Intraoperative FFP units**	1.026 (1.014-1.040)	**<0.001**		
**Postoperative RBC units[c]**	1.059 (1.044-1.074)	**<0.001**	1.008 (0.975–1.042)	0.636
**Postoperative FFP units[c]**	1.044 (1.034-1.054)	**<0.001**	**1.032 (1.008–1.056)**	**0.009**

Results given as hazard ratios (HR) with 95% confidence intervals (95% CI). Factors showing significant results in the univariable analysis were included in the multivariable Cox regression model. Abbreviations used: *CAR*, C-reactive protein-to-albumin ratio; *CRP*, C-reactive protein; *Alb*, albumin; *BMI*, body mass index; *labMELD*, laboratory model of end-stage liver disease score; *BAR*, balance of risk; *SOFT*, survival outcomes following liver transplantation; *OLT*, orthotopic liver transplantation; *ICU*, intensive care unit; *RRT*, renal replacement therapy; *RBC*, red blood cell; *FFP*, fresh frozen plasma. [a] Refers to Schlegel et al. [b] Refers to Rana et al. [c] Refers to blood products given during the first 7 days following OLT.

Supplementary Table S18. Association of perioperative factors with 5-year recipient survival in patients with EAD and 90-day mortality (incl. MELD score multivariable analysis)

**Perioperative factor**	**Univariable Cox regression analysis**		**Multivariable Cox regression analysis**	
	HR (95% CI)	*p*-value	HR (95% CI)	*p*-value
**CAR>41%**	2.695 (1.850-3.925)	<0.001	**1.951 (1.352–2.817)**	**<0.001**
**Donor age (years)**	1.007 (0.996-1.019)	0.218		
**Donor BMI**	0.984 (0.958-1.012)	0.258		
**Donor sex (male)**	0.848 (0.592-1.215)	0.370		
**Cause of donor death**	1.181 (0.987-1.413)	0.069		
**Allocation type**	1.358 (0.979-1.886)	0.067		
**Recipient age**	1.017 (0.999-1.036)	0.060		
**Recipient BMI**	0.998 (0.984-1.012)	0.796		
**Recipient sex (male)**	0.656 (0.454-0.947)	0.024		
**Etiology of liver disease**	0.964 (0.892-1.041)	0.349		
**labMELD**	1.045 (1.028-1.062)	<0.001	1.021 (0.998–1.043)	0.069
**BAR score**[a]	1.093 (1.059-1.128)	<0.001		
**SOFT score**[b]	1.050 (1.033-1.067)	<0.001		
**Recipient pre-OLT ICU (yes)**	2.767 (1.903-4.025)	<0.001		
**Recipient pre-OLT abdominal surgery (yes)**	0.935 (0.632-1.384)	0.737		
**Recipient inpatient pre-OLT (yes)**	1.864 (1.300-2.672)	<0.001	0.855 (0.542–1.350)	0.502
**Recipient pre-OLT encephalopathy (yes)**	1.475 (1.026-2.121)	0.036		
**Recipient pre-OLT ascites (yes)**	1.284 (0.889-1.854)	0.183		
**Pre-OLT renal failure (yes)**	1.800 (1.248-2.597)	0.002	1.263 (0.881–1.809)	0.204
**Pre-OLT RRT (yes)**	2.225 (1.464-3.383)	<0.001		
**Cold ischaemia time (minutes)**	1.000 (0.998-1.002)	0.805		
**Warm ischaemia time (minutes)**	1.020 (0.991-1.048)	0.241		
**Intraoperative RBC units**	1.044 (1.032-1.057)	<0.001		
**Intraoperative FFP units**	1.030 (1.017-1.043)	<0.001		
**Postoperative RBC units**[d]	1.057 (1.040-1.074)	<0.001	1.015 (0.985–1.046)	0.335
**Postoperative FFP units**[d]	1.040 (1.028-1.051)	0.024	**1.027 (1.006–1.048)**	**0.011**

Results given as hazard ratios (HR) with 95% confidence intervals (95% CI). Factors showing significant results in the univariable analysis were included in the multivariable Cox regression model. Abbreviations used: *CAR*, C-reactive protein-to-albumin ratio; *CRP*, C-reactive protein; *Alb*, albumin; *BMI*, body mass index; *labMELD*, laboratory model of end-stage liver disease score; *BAR*, balance of risk; *SOFT*, survival outcomes following liver transplantation; *OLT*, orthotopic liver transplantation; *ICU*, intensive care unit; *RRT*, renal replacement therapy; *RBC*, red blood cell; *FFP*, fresh frozen plasma. [a] Refers to Schlegel et al. [b] Refers to Rana et al. [c] Refers to blood products given during the first 7 days following OLT.

Supplementary Table S19. Association of perioperative factors with 5-year graft survival in patients with EAD and 90-day mortality (incl. MELD score multivariable analysis)

**Perioperative factor**	**Univariable Cox regression analysis**		**Multivariable Cox regression analysis**	
	HR (95% CI)	*p*-value	HR (95% CI)	*p*-value
**CAR>41%**	2.507 (1.762-3.566)	<0.001	**2.044 (1.374–3.041)**	**<0.001**
**Donor age (years)**	1.008 (0.997-1.019)	0.168		
**Donor BMI**	0.988 (0.963-1.013)	0.344		
**Donor sex (male)**	0.763 (0.543-1.072)	0.119		
**Cause of donor death**	1.070 (0.897-1.277)	0.454		
**Allocation type**	1.201 (0.886-1.628)	0.238		
**Recipient age**	1.007 (0.991-1.024)	0.367		
**Recipient BMI**	0.995 (0.977-1.014)	0.618		
**Recipient sex (male)**	0.696 (0.491-1.078)	0.186		
**Etiology of liver disease**	0.827 (0.924-1.065)	0.827		
**labMELD**	1.035 (1.019-1.051)	<0.001	1.021 (0.997–1.044)	0.085
**BAR score**[a]	1.069 (1.038-1.101)	<0.001		
**SOFT score**[b]	1.023 (1.023-1.057)	<0.001		
**Recipient pre-OLT ICU (yes)**	2.344 (1.632-3.366)	<0.001		
**Recipient pre-OLT abdominal surgery (yes)**	0.907 (0.626-1.315)	0.606		
**Recipient inpatient pre-OLT (yes)**	1.619 (1.150-2.888)	0.006	0.805 (0.494–1.313)	0.384
**Recipient pre-OLT encephalopathy (yes)**	1.173 (0.828-1.661)	0.369		
**Recipient pre-OLT ascites (yes)**	1.269 (0.897-1.796)	0.179		
**Pre-OLT renal failure (yes)**	1.502 (1.056-2.137)	0.024	0.932 (0.617-1.408)	0.356
**Pre-OLT RRT (yes)**	1.848 (1.224-2.789)	0.003		
**Cold ischaemia time (minutes)**	1.001 (0.999-1.002)	0.230		
**Warm ischaemia time (minutes)**	1.015 (0.998-1.033)	0.080		
**Intraoperative RBC units**	1.039 (1.028-1.051)	<0.001		
**Intraoperative FFP units**	1.025 (1.015-1.038)	<0.001		
**Postoperative RBC units**[d]	1.058 (1.043-1.073)	<0.001	1.014 (0.982–1.047)	0.402
**Postoperative FFP units**[d]	1.043 (1.032-1.053)	<0.001	**1.028 (1.005–1.052)**	**0.015**

Results given as hazard ratios (HR) with 95% confidence intervals (95% CI). Factors showing significant results in the univariable analysis were included in the multivariable Cox regression model. Abbreviations used: *CAR*, C-reactive protein-to-albumin ratio; *CRP*, C-reactive protein; *Alb*, albumin; *BMI*, body mass index; *labMELD*, laboratory model of end-stage liver disease score; *BAR*, balance of risk; *SOFT*, survival outcomes following liver transplantation; *OLT*, orthotopic liver transplantation; *ICU*, intensive care unit; *RRT*, renal replacement therapy; *RBC*, red blood cell; *FFP*, fresh frozen plasma. [a] Refers to Schlegel et al. [b] Refers to Rana et al. [c] Refers to blood products given during the first 7 days following OLT.

Supplementary Table S20. Association of perioperative factors with 3-year recipient survival in patients with EAD and 90-day mortality (incl. SOFT score multivariable analysis)

**Perioperative factor**	**Univariable Cox regression analysis**		**Multivariable Cox regression analysis**	
	HR (95% CI)	*p*-value	HR (95% CI)	*p*-value
**CAR>41%**	3.291 (2.141-5.058)	**<0.001**	**1.807 (1.237–2.638)**	**0.002**
**Donor age (years)**	1.004 (0.992-1.017)	0.483		
**Donor BMI**	0.975 (0.944-1.007)	0.129		
**Donor sex (male)**	0.782 (0.526-1.162)	0.223		
**Cause of donor death**	1.148 (0.942-1.400)	0.171		
**Allocation type**	1.053 (0.616-1.713)	0.816		
**Recipient age**	1.023 (0.985-1.053)	0.365		
**Recipient BMI**	0.999 (0.985-1.013)	0.880		
**Recipient sex (male)**	0.582 (0.334-1.021)	0.072		
**Etiology of liver disease**	0.978 (0.899-1.063)	0.595		
**labMELD**	1.056 (1.037-1.075)	**<0.001**		
**BAR score[a]**	1.116 (1.078-1.155)	**<0.001**		
**SOFT score[b]**	1.061 (1.043-1.080)	**<0.001**	1.023 (0.999–1.047)	0.065
**Recipient pre-OLT ICU (yes)**	3.526 (2.359-5.271)	**<0.001**		
**Recipient pre-OLT abdominal surgery (yes)**	0.978 (0.635-1.504)	0.918		
**Recipient inpatient pre-OLT (yes)**	2.529 (1.696-3.771)	**<0.001**	0.806 (0.497–1.310)	0.384
**Recipient pre-OLT encephalopathy (yes)**	1.773 (1.191-2.640)	**0.005**		
**Recipient pre-OLT ascites (yes)**	1.339 (0.892-2.010)	0.159		
**Pre-OLT renal failure (yes)**	2.441 (1.642-3.629)	**<0.001**	1.279 (0.864–1.893)	0.219
**Pre-OLT RRT (yes)**	2.880 (1.865-4.446)	**<0.001**		
**Cold ischemia time (minutes)**	1.001 (1.000-1.003)	**0.058**		
**Warm ischemia time (minutes)**	1.026 (1.008-1.045)	**0.005**		
**Intraoperative RBC units**	1.046 (1.043-1.059)	**<0.001**		
**Intraoperative FFP units**	1.032 (1.019-1.046)	**<0.001**		
**Postoperative RBC units[c]**	1.059 (1.042-1.076)	**<0.001**	1.015 (0.983–1.047)	0.363
**Postoperative FFP units[c]**	1.042 (1.030-1.053)	**<0.001**	**1.026 (1.004–1.048)**	**0.020**

Results given as hazard ratios (HR) with 95% confidence intervals (95% CI). Factors showing significant results in the univariable analysis were included in the multivariable Cox regression model. To avoid multicollinearity, certain variables found to be significantly different in the subgroup analysis were not included in the multivariable Cox regression analysis (e.g., postoperative RBC and FFP, etc.). Abbreviations used: *CAR*, C-reactive protein-to-albumin ratio; *CRP*, C-reactive protein; *Alb*, albumin; *BMI*, body mass index; *labMELD*, laboratory model of end-stage liver disease score; *BAR*, balance of risk; *SOFT*, survival outcomes following liver transplantation; *OLT*, orthotopic liver transplantation; *ICU*, intensive care unit; *RRT*, renal replacement therapy; *RBC*, red blood cell; *FFP*, fresh frozen plasma. [a] Refers to Schlegel et al. [b] Refers to Rana et al. [c] Refers to blood products given during the first 7 days following OLT.

Supplementary Table S21. Association of perioperative factors with 3-year graft survival in patients with EAD and 90-day mortality (incl. SOFT score multivariable analysis)

Perioperative factor	Univariable Cox regression analysis		Multivariable Cox regression analysis	
	R (95% CI)	*p*-value	HR (95% CI)	*p*-value
**CAR>41%**	2.907 (1.960-4.311)	**<0.001**	**1.983 (1.238–3.175)**	**0.004**
**Donor age (years)**	1.005 (0.993-1.016)	0.438		
**Donor BMI**	0.979 (0.950-1.008)	0.154		
**Donor sex (male)**	0.883 (0.507-1.533)	0.654		
**Cause of donor death**	1.028 (0.847-1.249)	0.777		
**Allocation type**	1.238 (0.884-1.732)	0.214		
**Recipient age**	1.013 (0.995-1.032)	0.162		
**Recipient BMI**	0.996 (0.978-1.015)	0.691		
**Recipient sex (male)**	0.579 (0.321-1.048)	0.079		
**Etiology of liver disease**	1.016 (0.942-1.096)	0.687		
**labMELD**	1.044 (1.027-1.062)	**<0.001**		
**BAR score[a]**	1.089 (1.054-1.124)	**<0.001**		
**SOFT score[b]**	1.049 (1.032-1.067)	**<0.001**	1.011 (0.983–1.040)	0.444
**Recipient pre-OLT ICU (yes)**	2.841 (1.936-4.168)	**<0.001**		
**Recipient pre-OLT abdominal surgery (yes)**	0.946 (0.632-1.416)	0.787		
**Recipient inpatient pre-OLT (yes)**	2.062 (1.423-2.989)	**<0.001**	1.069 (0.603–1.894)	0.820
**Recipient pre-OLT encephalopathy (yes)**	1.341 (0.921-1.953)	0.125		
**Recipient pre-OLT ascites (yes)**	1.357 (0.927-1.986)	0.116		
**Pre-OLT renal failure (yes)**	1.932 (1.328-2.809)	**<0.001**	1.050 (0.646–1.709)	0.844
**Pre-OLT RRT (yes)**	2.308 (1.512-3.525)	**<0.001**		
**Cold ischemia time (minutes)**	1.001 (0.999-1.002)	0.301		
**Warm ischemia time (minutes)**	1.013 (0.990-1.043)	0.219		
**Intraoperative RBC units**	1.040 (1.028-1.052)	**<0.001**		
**Intraoperative FFP units**	1.026 (1.014-1.040)	**<0.001**		
**Postoperative RBC units[c]**	1.059 (1.044-1.074)	**<0.001**	1.011 (0.976–1.047)	0.545
**Postoperative FFP units[c]**	1.044 (1.034-1.054)	**<0.001**	**1.029 (1.003–1.054)**	**0.027**

Results given as hazard ratios (HR) with 95% confidence intervals (95% CI). Factors showing significant results in the univariable analysis were included in the multivariable Cox regression model. Abbreviations used: *CAR*, C-reactive protein-to-albumin ratio; *CRP*, C-reactive protein; *Alb*, albumin; *BMI*, body mass index; *labMELD*, laboratory model of end-stage liver disease score; *BAR*, balance of risk; *SOFT*, survival outcomes following liver transplantation; *OLT*, orthotopic liver transplantation; *ICU*, intensive care unit; *RRT*, renal replacement therapy; *RBC*, red blood cell; *FFP*, fresh frozen plasma. [a] Refers to Schlegel et al. [b] Refers to Rana et al. [c] Refers to blood products given during the first 7 days following OLT.

Supplementary Table S22. Association of perioperative factors with 5-year recipient survival in patients with EAD and 90-day mortality (incl. SOFT score multivariable analysis)

Perioperative factor	Univariable Cox regression analysis		Multivariable Cox regression analysis	
	HR (95% CI)	*p*-value	HR (95% CI)	*p*-value
**CAR>41%**	2.695 (1.850-3.925)	<0.001	**1.830 (1.249–2.681)**	**0.002**
**Donor age (years)**	1.007 (0.996-1.019)	0.218		
**Donor BMI**	0.984 (0.958-1.012)	0.258		
**Donor sex (male)**	0.848 (0.592-1.215)	0.370		
**Cause of donor death**	1.181 (0.987-1.413)	0.069		
**Allocation type**	1.358 (0.979-1.886)	0.067		
**Recipient age**	1.017 (0.999-1.036)	0.060		
**Recipient BMI**	0.998 (0.984-1.012)	0.796		
**Recipient sex (male)**	0.656 (0.454-0.947)	0.024		
**Etiology of liver disease**	0.964 (0.892-1.041)	0.349		
**labMELD**	1.045 (1.028-1.062)	<0.001		
**BAR score**[a]	1.093 (1.059-1.128)	<0.001		
**SOFT score**[b]	1.050 (1.033-1.067)	<0.001	1.024 (0.997-1.049)	0.261
**Recipient pre-OLT ICU (yes)**	2.767 (1.903-4.025)	<0.001		
**Recipient pre-OLT abdominal surgery (yes)**	0.935 (0.632-1.384)	0.737		
**Recipient inpatient pre-OLT (yes)**	1.864 (1.300-2.672)	<0.001	0.779 (0.478–1.269)	0.315
**Recipient pre-OLT encephalopathy (yes)**	1.475 (1.026-2.121)	0.036		
**Recipient pre-OLT ascites (yes)**	1.284 (0.889-1.854)	0.183		
**Pre-OLT renal failure (yes)**	1.800 (1.248-2.597)	0.002	1.189 (0.806–1.753)	0.384
**Pre-OLT RRT (yes)**	2.225 (1.464-3.383)	<0.001		
**Cold ischaemia time (minutes)**	1.000 (0.998-1.002)	0.805		
**Warm ischaemia time (minutes)**	1.020 (0.991-1.048)	0.241		
**Intraoperative RBC units**	1.044 (1.032-1.057)	<0.001		
**Intraoperative FFP units**	1.030 (1.017-1.043)	<0.001		
**Postoperative RBC units**[d]	1.057 (1.040-1.074)	<0.001	1.017 (0.985–1.049)	0.302
**Postoperative FFP units**[d]	1.040 (1.028-1.051)	0.024	**1.024 (1.002–1.046)**	**0.034**

Results given as hazard ratios (HR) with 95% confidence intervals (95% CI). Factors showing significant results in the univariable analysis were included in the multivariable Cox regression model. Abbreviations used: *CAR*, C-reactive protein-to-albumin ratio; *CRP*, C-reactive protein; *Alb*, albumin; *BMI*, body mass index; *labMELD*, laboratory model of end-stage liver disease score; *BAR*, balance of risk; *SOFT*, survival outcomes following liver transplantation; *OLT*, orthotopic liver transplantation; *ICU*, intensive care unit; *RRT*, renal replacement therapy; *RBC*, red blood cell; *FFP*, fresh frozen plasma. [a] Refers to Schlegel et al. [b] Refers to Rana et al. [c] Refers to blood products given during the first 7 days following OLT.

Supplementary Table S23. Association of perioperative factors with 5-year graft survival in patients with EAD and 90-day mortality (incl. SOFT score multivariable analysis)

Perioperative factor	Univariable Cox regression analysis		Multivariable Cox regression analysis	
	HR (95% CI)	*p*-value	HR (95% CI)	*p*-value
**CAR>41%**	2.507 (1.762-3.566)	<0.001	**1.958 (1.293–2.964)**	**0.002**
**Donor age (years)**	1.008 (0.997-1.019)	0.168		
**Donor BMI**	0.988 (0.963-1.013)	0.344		
**Donor sex (male)**	0.763 (0.543-1.072)	0.119		
**Cause of donor death**	1.070 (0.897-1.277)	0.454		
**Allocation type**	1.201 (0.886-1.628)	0.238		
**Recipient age**	1.007 (0.991-1.024)	0.367		
**Recipient BMI**	0.995 (0.977-1.014)	0.618		
**Recipient sex (male)**	0.696 (0.491-1.078)	0.186		
**Etiology of liver disease**	0.827 (0.924-1.065)	0.827		
**labMELD**	1.035 (1.019-1.051)	<0.001		
**BAR score[a]**	1.069 (1.038-1.101)	<0.001		
**SOFT score[b]**	1.023 (1.023-1.057)	<0.001	1.011 (0.985–1.037)	0.428
**Recipient pre-OLT ICU (yes)**	2.344 (1.632-3.366)	<0.001		
**Recipient pre-OLT abdominal surgery (yes)**	0.907 (0.626-1.315)	0.606		
**Recipient inpatient pre-OLT (yes)**	1.619 (1.150-2.888)	0.006	0.856 (0.506–1.448)	0.563
**Recipient pre-OLT encephalopathy (yes)**	1.173 (0.828-1.661)	0.369		
**Recipient pre-OLT ascites (yes)**	1.269 (0.897-1.796)	0.179		
**Pre-OLT renal failure (yes)**	1.502 (1.056-2.137)	0.024	0.933 (0.618-1.408)	0.346
**Pre-OLT RRT (yes)**	1.848 (1.224-2.789)	0.003		
**Cold ischaemia time (minutes)**	1.001 (0.999-1.002)	0.230		
**Warm ischaemia time (minutes)**	1.015 (0.998-1.033)	0.080		
**Intraoperative RBC units**	1.039 (1.028-1.051)	<0.001		
**Intraoperative FFP units**	1.025 (1.015-1.038)	<0.001		
**Postoperative RBC units[d]**	1.058 (1.043-1.073)	<0.001	1.016 (0.983–1.051)	0.342
**Postoperative FFP units[d]**	1.043 (1.032-1.053)	<0.001	**1.026 (1.001–1.050)**	**0.039**

Results given as hazard ratios (HR) with 95% confidence intervals (95% CI). Factors showing significant results in the univariable analysis were included in the multivariable Cox regression model. Abbreviations used: *CAR*, C-reactive protein-to-albumin ratio; *CRP*, C-reactive protein; *Alb*, albumin; *BMI*, body mass index; *labMELD*, laboratory model of end-stage liver disease score; *BAR*, balance of risk; *SOFT*, survival outcomes following liver transplantation; *OLT*, orthotopic liver transplantation; *ICU*, intensive care unit; *RRT*, renal replacement therapy; *RBC*, red blood cell; *FFP*, fresh frozen plasma. [a] Refers to Schlegel et al. [b] Refers to Rana et al. [c] Refers to blood products given during the first 7 days following OLT.
